# Socioeconomic deprivation is inversely associated with measles incidence: a longitudinal small-area analysis, Germany, 2001 to 2017

**DOI:** 10.2807/1560-7917.ES.2021.26.17.1900755

**Published:** 2021-04-29

**Authors:** Sven Rohleder, Christian Stock, Kayvan Bozorgmehr

**Affiliations:** 1 Section for Health Equity Studies and Migration, Department of General Practice and Health Services Research, University Hospital Heidelberg, Heidelberg, Germany; 2 Department of Population Medicine and Health Services Research, School of Public Health, Bielefeld University, Bielefeld, Germany; 3 Institute of Medical Biometry and Informatics, University of Heidelberg, Heidelberg, Germany

**Keywords:** area deprivation, socioeconomic inequality, measles, Bayesian spatial analysis, infectious disease modelling

## Abstract

**Background:**

Although measles is endemic throughout the World Health Organization European Region, few studies have analysed socioeconomic inequalities and spatiotemporal variations in the disease’s incidence.

**Aim:**

To study the association between socioeconomic deprivation and measles incidence in Germany, while considering relevant demographic, spatial and temporal factors.

**Methods:**

We conducted a longitudinal small-area analysis using nationally representative linked data in 401 districts (2001–2017). We used spatiotemporal Bayesian regression models to assess the potential effect of area deprivation on measles incidence, adjusted for demographic and geographical factors, as well as spatial and temporal effects. We estimated risk ratios (RR) for deprivation quintiles (Q1–Q5), and district-specific adjusted relative risks (ARR) to assess the area-level risk profile of measles in Germany.

**Results:**

The risk of measles incidence in areas with lowest deprivation quintile (Q1) was 1.58 times higher (95% credible interval (CrI): 1.32–2.00) than in those with highest deprivation (Q5). Areas with medium-low (Q2), medium (Q3) and medium-high deprivation (Q4) had higher adjusted risks of measles relative to areas with highest deprivation (Q5) (RR: 1.23, 95%CrI: 0.99–1.51; 1.05, 95%CrI: 0.87–1.26 and 1.23, 95%CrI: 1.05–1.43, respectively). We identified 54 districts at medium-high risk for measles (ARR > 2) in Germany, of which 22 were at high risk (ARR > 3).

**Conclusion:**

Socioeconomic deprivation in Germany, one of Europe’s most populated countries, is inversely associated with measles incidence. This association persists after demographic and spatiotemporal factors are considered. The social, spatial and temporal patterns of elevated risk require targeted public health action and policy to address the complexity underlying measles epidemiology.

## Introduction

Measles is one of the most contagious infectious diseases, which approximately affected 6.7 million people and caused 110,000 deaths worldwide in 2017 [1]. Despite major reductions by ca 83% in measles global incidence between 2000 and 2017 (from 145 to 25 notified cases per million) [1], the disease is still considered a serious vaccine-preventable cause of morbidity and mortality worldwide [2].

Measles currently remains endemic in all the World Health Organization (WHO) Regions, and immunisation programmes are challenged in many countries, with decline or stagnation of vaccination rates [3]. Estimates from the WHO for 2018 show that the global coverage with the first dose of measles vaccine in children was 86%, however, less than 69% of children received the recommended second dose [4]. A high number of measles cases have been reported between 2017 and 2019 in the WHO European Region and measles remains endemic in 10 European countries, including Belgium, Bosnia and Herzegovina, France, Georgia, Germany, Italy, Romania, Russia, Serbia and Ukraine [5]. As a consequence of recurrent outbreaks and continuing occurrence of infections, the WHO recorded an increase of measles incidence in the WHO European Region by almost 300% until July 2019 compared with 2018 [6].

Measles incidence and transmission in a population is effectively prevented by vaccination of at least 95% of individuals with two doses of measles vaccine to ensure herd immunity, i.e. protection of everyone including those who cannot be immunised [7]. Delivering this intervention remains, however, a challenge even for strong healthcare systems. Moreover, measles is a very dynamic infectious disease and its distribution is closely linked to geographical and time-dependent factors as well as to demographic and socioeconomic determinants [2,8]. The complex interplay between measles incidence and socioeconomic factors (i.e. income, education and occupation) is yet not fully understood. Public health interventions thus fall short of addressing structural factors that may intersect and operate at both individual and small-area level.

In the European Region, vaccination coverage is suboptimal at subnational level and immunity gaps exist, leaving marginalised groups or elderly persons vulnerable to infection [5]. For example, in Germany, regional vaccination rates vary considerably, especially with lower rates in southern Germany. In addition, over time, there are signs of regression in vaccination coverage in all German states until 2018 [9]. Correspondingly, highest incidence rates of measles virus infection were observed in southern Germany [10]. In terms of socioeconomic status (SES), childhood vaccination against measles is lowest in populations with highest SES in the United Kingdom (UK) and Germany [11,12]. In Germany, for example, children and adolescents in families with low SES show higher vaccination coverage rates for the first dose of the mumps, measles and rubella (MMR) vaccine compared with those from families with high SES, while no difference between children with different SES exists for the second dose. This observation supports the assumption that, generally, parents with high SES who decide to get their child vaccinated are more likely to complete a vaccination series than parents from families with low SES [13]. The role of socioeconomic inequalities in vaccination coverage variation have been extensively examined in previous studies in Europe based on individual-level (e.g. educational attainment, income, or occupation) and area-level measures of SES (e.g. area deprivation) [14,15]. Although these studies report mixed results regarding the magnitude and direction of socioeconomic inequalities in vaccination coverage, they demonstrate that considering these factors for the development of effective public health strategies against measles is crucial.

The evidence on the association between area-level socioeconomic status and measles incidence is scarce and predominantly characterised by cross-sectional study designs. Only few existing studies consider spatiotemporal dependencies of measles and area deprivation: two studies report higher risk of measles incidence in regions with lower deprivation level in Italy [16] and England [17]; another study from China showed higher measles burden in cities, which are more economically developed [18].

While the association between socioeconomic factors and vaccination coverage has been shown by several studies, very few studies have directly analysed the relationship between socioeconomic inequalities and the incidence of measles. Furthermore, the predominantly cross-sectional nature of existing studies precludes important considerations of spatiotemporal dynamics of measles distribution and patterns in economic, social, and demographic factors. Using the example of Germany, the most populated country of the European Union, the aim of our study was to analyse the spatiotemporal association between socioeconomic deprivation and measles incidence considering relevant demographic and geographical factors at the district level from 2001 to 2017.

## Methods

### Study design

We conducted a longitudinal small-area analysis covering 401 German districts, corresponding to Nomenclature of territorial units for statistics (NUTS 3), to assess the association between area deprivation and measles incidence from 2001 to 2017, considering also the potential effects of relevant socio-demographic, and spatial as well as temporal factors. We further derived the spatial risk-profile of individual districts and mapped their posterior probability of exceeding risk thresholds.

### Data sources and data preparation

We combined nationally representative district data from four different sources including data on measles incidences, area deprivation, population statistics and geographical data. Yearly data on incident measles virus infections (2001 to 2017), stratified by sex and age groups (0–14, 15–29, 30–44, 45–64, ≥ 65 years) were obtained from the national surveillance statistics regarding notified infectious diseases, which are compiled by the Robert-Koch Institute (RKI), the national public health agency in Germany [19]. Measles cases with unknown sex or age were excluded from the analysis. We used all measles records notified to the RKI, including those with laboratory diagnostic evidence but unfulfilled or unknown clinical picture. The German Index of Socioeconomic Deprivation (GISD), a contextual composite index measure on area deprivation developed by the RKI, was used as exposure variable of primary interest [20]. Area deprivation is here assumed to reflect the SES of a geographical unit and can be used to assess the extent of area-level social inequalities in health [20]. The GISD consists of three domains (education, occupation, and income of the population), builds on nationally representative data of the Federal Statistics Office, and is constructed for 402 districts in Germany in 5-year intervals since 1998. We adopted the versions of the GISD for following defined periods: GISD 1998 (2001 to 2002), GISD 2003 (2003 to 2007), GISD 2008 (2008 to 2011) and GISD 2012 (2012 to 2017). As a result of an administrative district reform in 2016 two districts in Lower Saxony were merged to one district. Considering this, we calculated population-weighted scores based on the underlying GISD values, and classified the GISD-scores of the 401 districts into quintiles. Districts with the lowest socioeconomic deprivation (highest SES) were assigned to the lowest quintile (Q1), those with highest deprivation (lowest SES) were assigned to the highest quintile (Q5).

We obtained population statistics on the district-level from a database of the system of social reporting in official statistics for 2001 to 2017; these were used to calculate expected incidences of measles virus infections and the proportion of non-nationals among the districts’ population [21]. Data on population size was missing in three districts for the years 2001 to 2010 and in one district for 2001 to 2008. Missing data (0.5%) were linearly substituted.

Geographical data were obtained from the Federal Agency of Cartography and Geodesy [22].

In addition to deprivation, we considered sex and age groups, proportion of non-nationals, federal state and geographical factors, as well as (structured and unstructured) spatial and temporal effects as potential predictors of district-specific measles incidence.

### Statistical analysis

We calculated district-level standardised incidence ratios (SIR) stratified by sex and age group as the ratio of observed to expected measles cases. Observed incidences for each stratum were calculated by the period mean over 17 years of notified measles cases over the population size in each stratum at district-level. Expected incidences for each stratum were calculated using the national-level stratified mean incidence rate of measles in Germany multiplied with the respective district population size. In addition, we computed mean SIR for age groups with 95% confidence intervals (CI) on national level.

Furthermore, we examined SIRs per year for the GISD quintiles with corresponding 95%CI, and calculated population-weighted means (WM) to pool SIR over timespan.

We then examined the association between area deprivation and the incidence of measles using negative binomial Bayesian spatiotemporal regression models fitted by the integrated nested Laplace approximation (INLA) approach [23,24]. Due to excess zeros in the observed incident case numbers (extra-Poisson variation in the intercept-only models), we used zero-inflated negative binomial models. We fitted 14 models by iteratively adjusting them for explanatory variables, and gradually increasing their complexity (Supplementary Tables S1, S4 and S5). We adjusted the effect of area deprivation on measles incidence for district population size, sex and age groups (0–14, 15–29, 30–44, 45–64, ≥ 65 years), proportion of non-nationals, temporal effects, geographical factors (federal states effects, or north–south–east–west effects), structured and unstructured spatial effects, and parametric temporal trend, or dynamic temporal trend. The considered spatial and temporal effects also allow capturing potential outbreak dynamics of measles incidence within the model. The Watanabe–Akaike information criterion (WAIC) informed our model selection for the best model fit; see Supplementary Tables S1 to S5 for detailed information on model selection.

Using the results of the best fit model, we then estimated risk ratios (RR) and corresponding 95% credible intervals (CrI) to assess the effects of area deprivation (quintiles), sex and age groups, proportion of non-nationals (quintiles) and geographical factors (north–south–east–west effects). To analyse spatial risk patterns of measles virus infections, we first calculated district-specific adjusted relative risks (ARR), a measure that is adjusted for the included fixed and random effects, and combines the spatially structured and unstructured effects [25,26]. Secondly, we computed the Bayesian exceedance probability for defined ARR thresholds (ARR > 1, 1.5, 2, or 3, respectively) in order to identify areas with moderate (more than twofold) or high (more than threefold) elevated risk. We adopted a stricter interpretation of the Richardson criterion (suggesting a 70–80% posterior probability), by setting the cut-off for elevated risk at a posterior probability of ≥ 80% [27]. We further assessed the posterior temporal main effect (TME) by combining the structured and unstructured time effects. We used the R language and environments for statistical computing (V.3.6.0) for the analysis and the R package R-INLA [24] to fit spatiotemporal Bayesian models.

### Regression model specification

We specified Bayesian zero-inflated negative binomial spatiotemporal models. For each district *i* (*i* = 1,…,401) and *t^th^* time point (*t *= 1,…,17) it was assumed that measles incidence *Y = y_it_* follows a negative binomial (*NB*) distribution, 

$$g\left(y_{it}\right)=\;p\left({Y=y}_{it}\right)=NB(n_{it},\;p_{it})=\;\frac{\Gamma \left(y_{it}+n_{it}\right)}{\Gamma \left(n_{it}\right)\Gamma \left(y_{it}+1\right)}p_{it}^{n_{it}}{\left(1-p_{it}\right)}^{y_{it}}$$,

where Γ(∙) is the gamma function, *n_it_* is the number of successful trials (dispersion parameter) and *p_it _*the probability of success in each trial. The mean *μ*_it_ and variance *σ^2^_it_* of *y_it_* were 

$$\mu_{it}=n_{it}\frac{1-p_{it}}{p_{it}}\;,\;{\sigma^{2}}_{it}=\mu_{it}\left(1+\frac{\mu_{it}}{n_{it}}\right)\;,$$

with link function *μ_it_*= *E_it_e^ηit ^*and hyperparameter for dispersion size *n_it_*= *e^Θ1^*. *E_it_*represents the expected number of cases and log(*E_it_*) is the offset of *η_it_*. *Θ_1_* is a set of parameters for the dispersion size with given prior and initial value.

Assuming not-structural zeros in *i^th^* district (sample zeros) with zero-inflation parameter 

$$\pi_{0}=\frac{e^{\theta_{2}}}{1+e^{\theta_{2}}}$$,

the probability function for *y_it_* is 

$$p\left(y_{it}|\pi_{0}\right)=\pi_{0}\times 1_{\left[y_{it}=0\right]}+(1-\pi_{0})\times g\left(y_{it}\right)$$.

*Θ_2_* is a set of parameters for *π_0_* with given prior and initial value.

Conditional on* y_it _*not being a structural zero, where *μ_it_* is defined in terms of rate *r_it_* and *E_it _*with *μ_it_*= *E_it_r_it_* **and log(*r_it_*)* = η_it_, *the model**that fit the data best was then specified considering the linear predictor *η_it_*defined on a logarithmic scale: 

$$\eta_{it}=\alpha +\sum_{k=1,\;j=2}^{k=4,j=5}\beta_{k}{GISDQ}_{jit}+\beta_{5}{Sex}_{it}+\sum_{k=6,\;j=2}^{k=9,j=5}\beta_{k}{Age}_{jit}+\sum_{k=10,\;j=2}^{k=13,j=5}\beta_{k}{\%NonN}_{jit}+\sum_{k=14,\;j=2}^{k=16,j=4}\beta_{k}{GeoS}_{jit}+u_{i}+\upsilon_{i}+\gamma_{t}+\phi_{t}.$$

*α* is the intercept, *β_k _*are the coefficients of the effects in GISD quintiles (*GISDQ_jit_*), sex (*Sex_it_*), age groups (*Age_jit_*), quintiles of the proportion of non-nationals (*%NonN_jit_*), and north–south–east–west effects (*GeoS_jit_*). *u_i_* is a spatially structured random effect modelled using intrinsic conditional auto-regression (Besag model) and *υ_i_* a spatially unstructured effect modelled using exchangeability among the areas (independent and identically distributed, iid). The incorporation of the random effects *u_i _*and *υ_i_* in one model is also known as Besag–York–Mollié model

$$x=\left(\begin{array}{c} \upsilon_{i}+u_{i}\\ u_{i} \end{array}\right)$$.

A nonparametric dynamic temporal trend was modelled to incorporate a structured temporal effect *γ_t_*, modelled using random walk of order two, and an unstructured temporal effect *ϕ_t_*, modelled exchangeable using iid.

The ARR is the posterior mean of the marginal posterior distribution of the spatial structured and unstructured random-effects, defined by *ARR_i_*= *e^ui+υi^*. Analogously, the TME is the posterior mean effect of the temporal structured and unstructured effects and is defined by *TME_t_*= e*^γt^* ^+^ *^ϕt^*. 

Default settings of the INLA algorithm were used to specify the prior distributions of the considered effects and hyperparameters. A Gaussian prior with mean 0 and precision 10 ^− 3^ was used for fixed-effects with *β_x_*~* Gaussian* (0, 10^−3^). For random effects, a logGamma prior was implemented with equivalent precision to *τ *~* Gamma* (1, 5^−5^).The prior of the overdispersion parameter based on a penalised complexity prior (pc.mgamma prior) including a parameter of value seven and initial value of 2.30258509299405. For the zero-inflation parameter a Gaussian prior was used with *π* ~ *Gaussian*(−1, 0.2) and initial value of −1.

### Ethical statement

The study used aggregated secondary data. An ethical approval was not required.

## Results

Of 26,109 measles virus infections that were notified in Germany between 2001 and 2017, 51% (n = 13,279) were among male, and 65% (n = 17,071) among children or adolescents aged between 0 and 14 years. The median district population size in terms of those aged > 17 years was 149,959 with corresponding inter-quartile range (IQR) 135,110.

**Figure 1:**
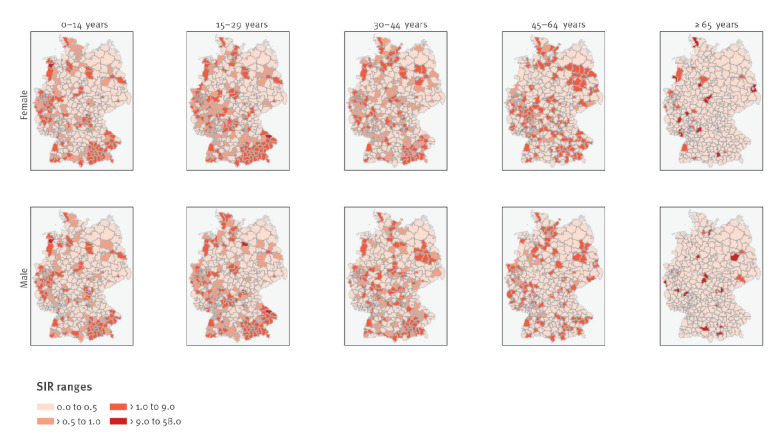
Standardised incidence ratios of measles virus infections in districts by sex and age groups, Germany, 2001–2017 (n = 401 districts)

Mean district-specific SIRs were 1.08 (95%CI: 0.84–1.33) among children (0 to 14 years), 0.93 (95%CI: 0.73–1.03) among adolescents and young adults (15 to 29 years), 0.67 (95%CI: 0.58–0.76) among middle aged (30 to 44 years), 0.73 (95%CI: 0.65–0.79) among those aged 45 to 64 years, and 0.99 (95%CI: 0.68–1.30) among the elderly (≥ 65 years). Elevated SIR (> 1) were more concentrated in southern Bavaria, in the Ruhr district in western Germany, in Berlin and its periphery districts, and in a few districts in northern and central Germany (Figure 1).

SIR: standardised incidence ratios.

SIR of measles virus infections stratified by sex and age groups (ratio of observed and expected incidences as period mean over 17 years). SIR > 1 means that observed incidence were higher than expected.

**Figure 2:**
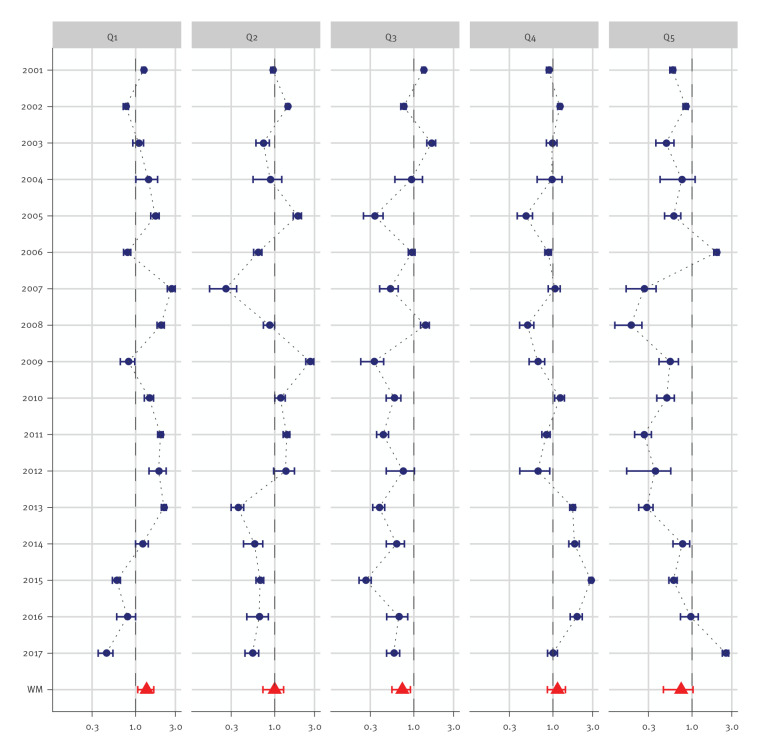
Distribution over the study period of the annual SIR of measles virus infections by deprivation quintiles and, for each quintile, the population-weighted mean of SIR over the period, Germany, 2001–2017

The largest average SIRs were found in areas with lowest socioeconomic deprivation (Q1 WM SIR of 1.35 and 95%CI: 1.06–1.65), and in districts with medium-high deprivation (Q4 WM SIR: 1.14; 95%CI: 0.86–1.41) (Figure 2). SIRs lower than 1, indicating lower observed incidences for measles virus infections than expected, were found in areas with medium (Q3 WM SIR: 0.72; 95%CI: 0.55–0.91) and highest socioeconomic deprivation (Q5 WM SIR: 0.74; 95%CI: 0.45–1.03). However, regarding the most recent years 2015 to 2017, SIRs in lowest deprived districts (Q1) declined and increased in most deprived districts (Q5).

Q: quintile; SIR: standardised incidence ratios; WM: weighted mean.

SIR of measles virus infections (ratio of observed and expected incidences) stratified by deprivation Q1 (lowest area deprivation) to Q5 (highest area deprivation). The population-WM of SIRs pooled over 17 years is also shown per quintile. SIR > 1 means that observed incidence were higher than expected.

**Figure 3:**
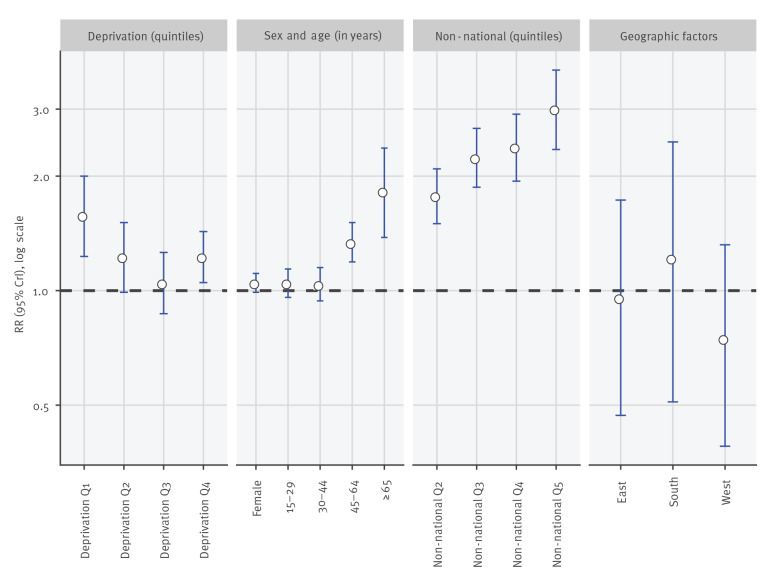
Bayesian regression adjusted risk-ratios for measles virus infections (fixed-effects), by deprivation, sex, age, non-nationals and geographical factors, Germany, 2001–2017 (n = 401 districts)

After full adjustment for fixed and random effects, socioeconomic deprivation tended to be inversely associated with measles incidence: the lower the area deprivation, the higher the area-level risk of measles virus infections (Figure 3). Compared with areas with highest deprivation (Q5), we found a decreasing risk gradient for measles virus infections from low (Q1) to medium deprived areas (Q3), but slightly elevated estimates for medium-high deprivation (Q4) resulting in a U-shaped association. The risk of measles virus infection in areas with lowest deprivation (Q1) was 1.58 times higher (95%CrI: 1.32–2.00) than in those with highest deprivation (Q5). Areas with medium-low (Q2), medium (Q3), and medium-high deprivation (Q4) had higher adjusted risks of measles relative to areas with highest deprivation (Q5), with RR of 1.23 (95%CrI: 0.99–1.51), 1.05 (95%CrI: 0.87–1.26), and 1.23 (95%CrI: 1.05–1.43), respectively.

CrI: credible intervals; Q: quintiles; RR: risk ratios.

RR and 95%CrI for measles virus infections associated with deprivation Qs (lowest deprivation Q5 as reference), age (0–14 years age group as reference) and sex (male as reference), Qs of the proportion of non-nationals (districts with lowest proportion of non-nationals as reference (Q1)), and geographical factors (northern Germany as reference). RRs greater than 1 (black dotted line) mean an elevated risk for measles virus infections.

Females showed only slightly higher risk for measles virus infection than males (RR: 1.05; 0.99–1.11). Higher risks of measles virus infections were found in adults aged 45 to 64 (RR: 1.34; 95%CrI: 1.19–1.51) and ≥ 65 (RR: 1.82; 95%CrI: 1.38–2.37) years compared with children aged 0 to 14 years. In populations aged 15 to 29 (RR: 1.05; 95%CrI: 0.96–1.14) or 30 to 44 (RR: 1.04; 95%CrI: 0.94–1.15) years marginally higher risks occurred.

Districts with a higher proportion of non-nationals among the population showed higher risk of measles: the risk of measles virus infections in districts with medium-low (%NNQ2), medium (%NNQ3), medium-high (%NNQ4) and highest (%NNQ5) proportion of non-nationals was 1.78 (95%CrI: 1.50–2.09), 2.25 (95%CrI: 1.87–2.67), 2.39 (95%CrI: 1.94–2.91), and 3.01 (95%CrI: 2.35–3.80) times the risk in districts with lowest proportion of non-nationals (%NNQ1), respectively.

As for geographical factors, southern Germany showed a slight tendency of higher risk relative to northern Germany (RR: 1.22; 95%CrI: 0.51–2.46), while western Germany (RR: 0.75; 95%CrI: 0.39–1.32) showed slight tendencies to lower risk.

We observed recurrent but progressing stabilised temporal peaks in risks estimates from 2001 to 2017 in Germany (Supplementary Figures S1 and S2), where highest risks were apparent in 2001 (TME: 8.00; 95%CrI: 1.37–26.19) and lowest in 2004 (TME: 0.24; 95%CrI: 0.07–0.58). From 2005 onwards, fluctuations with elevated risk (TME > 1) occurred almost every 2 years, with highest risks in the years 2006 (TME: 2.21; 95%CrI: 0.97–4.34), 2011 (TME: 2.04; 95%CrI: 1.02–3.64), 2013 (TME: 1.98; 95%CrI: 0.74–4.29) and 2015 (TME: 2.44; 95%CrI: 0.64–6.53); see Supplementary Figures S1 and S2.

**Figure 4:**
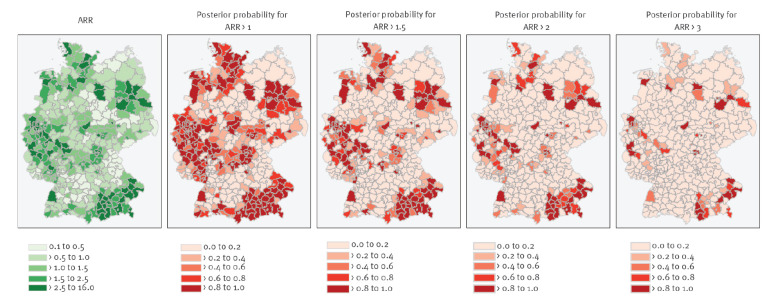
Mapping of Bayesian regression adjusted spatial relative risk and posterior probability of exceeding adjusted-spatial-relative-risk threshold in districts, Germany, 2001–2017 (n = 401 districts)

**Figure 5:**
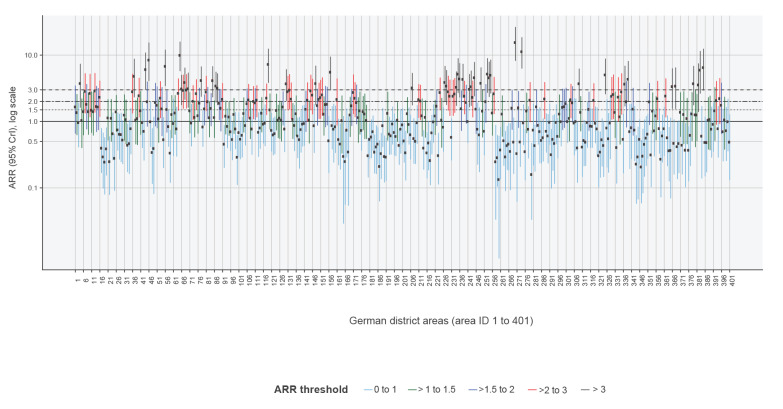
Bayesian regression adjusted spatial relative risk and exceedance thresholds, Germany, 2001–2017 (n = 401 districts)

ARR: adjusted spatial relative risk; CrI: credible interval; ID: identity.

The spatial risk profile (Figure 4 and 5) of measles virus infections was overall higher in south-eastern Bavaria in southern Germany, in the Ruhr area in North-Rhine Westphalia in western Germany, in Schleswig-Holstein in northern Germany, and in Berlin and its periphery districts in eastern Germany (see Supplementary Figure S4 for spatial risk profile stratified by federal states). Isolated districts with increased risks were detected in Rhineland-Palatinate, and Hesse in western and central Germany (see our interactive map in Supplement S1 for detailed information). We identified 54 districts with medium-high risk for measles virus infections and an ARR exceeding 2 (with a posterior probability equal to or greater than 80%). Of these, an ARR of 3 was exceeded in 22 districts, which were thus at high risk of measles virus infections (see Supplementary Tables S6 to S8 for full list of districts, ARR and posterior probability estimates).

ARR: adjusted spatial relative risk.

District-specific ARR for measles virus infections in Germany. ARR > 1 means a higher areal risk in district for measles virus infections. District with a posterior probability ≥ 80% for the thresholds of ARR > 2 and ARR > 3 were defined as medium and high risk for measles virus infections, respectively.

District-specific ARR and corresponding 95%CrI for measles virus infections of the 401 districts in Germany, coloured according to the ARR thresholds. ARR > 1 means a higher areal risk in district for measles virus infections.

## Discussion

Areas with lower socioeconomic deprivation in Germany tend to be at higher risk for measles incidence, adjusted for population characteristics (age, sex, proportion of non-nationals) and spatiotemporal factors. Of 401 districts, we identified 54 districts at medium-high risk, and among those 22 districts at high risk for measles over a period of 17 years. The highest risk for measles was primarily concentrated in southern Germany (especially in south Bavaria), as well as in the Ruhr-metropole and the periphery of Berlin, which is socioeconomically less deprived than Berlin. Those aged 45–64 years and ≥ 65 years were at highest risk of measles. Additionally, areas with high proportion of non-nationals showed higher adjusted risk of measles incidence. The longitudinal analysis identified important patterns: recurrent temporal peaks of measles risk are closely linked to higher yearly incidences.

Our study provides robust evidence that areas with higher SES (i.e. lower socioeconomic deprivation) are at considerably higher risk of measles than areas with lower SES (i.e. higher socioeconomic deprivation). These findings are consistent with an analysis showing that spatial clusters of low vaccination rates of measles in Germany are characterised by higher SES (measured by area-level measures of unemployment, household income, welfare, and a socioeconomic index) [28]. In Germany, a large proportion (13%) of the population has a substantial scepticism against vaccines [29], which may relate to inappropriate information on vaccine effects or side effects [30]. Although we had no data on preferences for or against vaccines, our analysis shows that better-off districts have considerably higher risk for measles virus infections than less privileged districts, despite available services, and adjusted for differences in demographic population characteristics.

Our results have three important implications for measles control policies. Firstly, targeted public health communication and outreach vaccination programmes towards districts at higher risk of measles incidence appears necessary. In Germany, vaccination is delivered predominantly by individual primary care physicians and paediatricians within a walk-in model of services [31]. National recommendations and vaccination goals are in place, but the health system has a strong focus on individualised medicine [32] with only few proactive outreach programmes to close immunisation gaps among the population (such as regular childhood examinations, school entry health checks and information campaigns). While primary care services are distributed relatively equitably across the 401 districts [33], the system’s gate-keeper function has been assessed as weak [31], and patients are not obliged to register with a primary healthcare centre. The public health services are usually not involved in provision of vaccination services to the general population except in cases of outbreaks. A national action plan for immunisation is in place with multiple actors involved at different levels of the decentralised health system, but the complex governance of immunisation activities and shortfalls in monitoring vaccination coverage at small-area level create challenges for attempts to proactively close immunisation gaps. Targeting districts with higher small-area risk of measles incidence may help to overcome these challenges, by paying specific or additional attention to such areas and seeking for tailored public health approaches.

Secondly, public health response to measles control should, in addition to those aged 0–14 years, incorporate 45−64 year olds and elderly population groups (≥ 65 years), e.g. through setting-based interventions in the work place for those in working age, or through community-based interventions and vaccination programmes for elderly people. In March 2020, the German Government passed a legislation to implement compulsory vaccination against measles in children attending schools and residents of nursing homes [34]. While this covers some of the groups identified at high risk in our study, it is likely to miss some of the above-mentioned population groups at high risk for measles virus infections.

Thirdly, low-threshold healthcare services are required to reach out to migrants in districts with high proportions of non-nationals. Migrants in Germany show lower utilisation of primary care services [35], and barriers exist in access to healthcare services, e.g. for European Union nationals (the largest migrant group) with respect to limitations of the European Health Insurance Card in providing preventive primary care services [36]. Vaccination against measles (and other infectious diseases) is part of the service package to which asylum seekers are entitled to in Germany. However, empirical assessments of the healthcare systems show that such services in reception centres and large accommodation centres are not routinely offered, or are often insufficiently provided [37]. We argue that in addition to nationwide standards for reception of and infectious disease control among asylum seekers [37,38], mandating public health services to deliver coordinated and tailored low-threshold vaccination programmes among regular migrants would be an important contribution to effective measles control.

Our study has several strengths. We used nationally representative data on measles virus infections, population characteristics, and area deprivation over 17 years to study spatiotemporal dynamics of measles incidence using Bayesian modelling techniques. The nationwide longitudinal analysis allowed estimation of fixed effects of exposure and co-variables, as well as structured and unstructured spatial and temporal random-effects on measles incidence. As shown by a recent global analysis of measles incidence, spatial units identified to be at higher risk of measles are very likely to show higher measles incidences as well in the future [39]. As such, the districts identified by our study as being at medium-high and high risk for measles are likely to be those disproportionately contributing to measles incidence in the future (despite the partially wide CrIs which are due to the high within-district variability of measles incidence over time; see Supplement). Our study also contributes to the yet small body of evidence on socioeconomic inequalities and infectious diseases:  studies have been conducted in Australia [40] and Sweden [41] showing a classical socioeconomic gradient in infectious disease incidence with higher disease burden among groups or areas with lower SES. Our study adds to this evidence, indicating that socioeconomic inequalities are not naturally always to the disadvantage of the less-privileged, and that associations may be inverse. This adds to the complexity of the relationship between socioeconomic factors and infectious diseases [42].

Our study is limited by the lack of individual-level variables for SES of notified measles virus infections, as such information is not reported. While the associations identified at population- and district-level (between lower area deprivation and higher risk of measles) are valid, the conclusion that individuals with higher SES are at higher risk of measles would not be appropriate (ecological fallacy). It is, at least theoretically, possible that the inverse is the case, and that individuals with lower SES living in better-off areas contribute to the disproportionately high incidence of measles in these areas. However, we think that this scenario is very implausible, and that overemphasising individual-level SES over area-level measures is prone to atomistic fallacy. Multilevel studies, linking individual- with area-level measures of SES, are required to disentangle such potentially existing complex relationships. Another limitation of our study is due to the nature of national routine surveillance systems. The official notification data are systematically validated following a standardised process, however, potential underreporting of the included surveillance data may be present [10]. Furthermore, potential within-district heterogeneity of the index measure for socioeconomic deprivation could be present in the data due to the effect of administrative boundaries (modifiable areal unit problem). However, the GISD that we used as measure for area-level deprivation is based on weighted indicators for the three dimensions education, occupation and income. Those indicators are predominantly proportions of the district population size. Therefore, within the GISD methodology, weighting methods were applied to increase the socioeconomic homogeneity on district level [43]. Defining a set of areas of consistent size (similar to “*Super Output Areas*” in the UK) for the GISD as well as for the notified infectious disease data in Germany would be a helpful way to reduce within-district heterogeneity for future studies [44].

### Conclusion

Socioeconomic deprivation in Germany, one of the most populated European countries, is inversely associated with measles incidence, with higher risks for measles virus infections being concentrated in areas with highest SES. Targeted vaccination programmes and public health policy, especially in districts with lower area deprivation, among middle-aged and elderly population groups, as well as in districts with high proportions of non-nationals seem required to enhance measles control strategies in Germany. Although our findings are context-specific, similar patterns of risk may exist in other European countries with endemic measles. Our findings contribute to current global and national debates on measles elimination strategies, and demonstrate the value of spatial modelling techniques in identifying socioeconomic determinants and spatial risk patterns of measles for public health actions.

## Supplementary Data

1207000 Supplement

## Supplementary Data

5605000 Supplement S1
