# Supplementary material for: Socioeconomic deprivation is inversely associated with measles incidence: a longitudinal small-area analysis, Germany, 2001 to 2017
Source: Euro Surveill. 2021 Apr 29;26(17):1900755. doi: 10.2807/1560-7917.ES.2021.26.17.1900755 (PMC8086244; doi:10.2807/1560-7917.ES.2021.26.17.1900755)
Supplement: Supplement [file 1900755_Supplement.pdf]

Supplementary material

"This supplementary material is hosted by Eurosurveillance as supporting information alongside the article "Socioeconomic deprivation is inversely associated with measles incidence: a longitudinal small-area analysis, Germany, 2001 to 2017", on behalf of the authors, who remain responsible for the accuracy and appropriateness of the content. The same standards for ethics, copyright, attributions and permissions as for the article apply. Supplements are not edited by Eurosurveillance and the journal is not responsible for the maintenance of any links or email addresses provided therein."

Model selection process

We first fitted two intercept-only models to test for potential overdispersion and zero-inflation; Poisson intercept-only model (M0_P), and negative binomial intercept-only model adjusted for zero-inflation (M0_znbin). In order to select the model that fits the data best, we then specified 14 Bayesian zero-inflated negative binomial regression models M1 to M14 (Table S.1) that became gradually more complex. We compared the respective model fit using the Watanabe–Akaike information criterion (WAIC) and selected the model with the lowest WAIC.

A spatio-temporal model that includes a Besag-York-Mollié model (BYM) and a non-parametric dynamical temporal trend (M13) fit the data best, and its results were prepared for the main manuscript.

Table S.2 (M0 to M7) and table S.3 (M8 to M14) comprise the results of the fitted models.

Table S1: Model formulas

Model	Formula
**M0_P**<br>Poisson	$\eta_{it} = \alpha$
**M0_znbin**<br>zero-inflated negative binomial (zbin)	$\eta_{it} = \alpha$
**M1**<br>zbin, Besag-York-Mollié (BYM)	$\eta_{it} = \alpha + u_i + v_i$
**M2**<br>znbin,	$\eta_{it} = \alpha \sum_{k=1,j=2}^{k=4,j=5} \beta_k GISDQ_{jit} + u_i + v_i$
**M3**<br>znbin, BYM	$\eta_{it} = \alpha + \sum_{k=1,j=2}^{k=4,j=5} \beta_k GISDQ_{jit} + \beta_5 Sex_{it} + u_i + v_i$
**M4**<br>znbin, BYM	$\eta_{it} = \alpha + \sum_{k=1,j=2}^{k=4,j=5} \beta_k GISDQ_{jit} + \beta_5 Sex_{it} + \sum_{k=6,j=2}^{k=9,j=5} \beta_k Age_{jit} + u_i + v_i$
**M5**<br>znbin, BYM	$\eta_{it} = \alpha + \sum_{k=1,j=2}^{k=4,j=5} \beta_k GISDQ_{jit} + \beta_5 Sex_{it} + \sum_{k=6,j=2}^{k=9,j=5} \beta_k Age_{jit} + \sum_{k=10,j=2}^{k=13,j=5} \beta_k \%NonN_{jit} + u_i + v_i$
**M6**<br>znbin, BYM	$\eta_{it} = \alpha + \sum_{k=1,j=2}^{k=4,j=5} \beta_k GISDQ_{jit} + \beta_5 Sex_{it} + \sum_{k=6,j=2}^{k=9,j=5} \beta_k Age_{jit} + \sum_{k=10,j=2}^{k=13,j=5} \beta_k \%NonN_{jit} + \sum_{k=14,j=2}^{k=29,j=17} \beta_k Year_{ji} + u_i + v_i$
**M7**<br>znbin, BYM	$\eta_{it} = \alpha + \sum_{k=1,j=2}^{k=4,j=5} \beta_k GISDQ_{jit} + \beta_5 Sex_{it} + \sum_{k=6,j=2}^{k=9,j=5} \beta_k Age_{jit} + \sum_{k=10,j=2}^{k=13,j=5} \beta_k \%NonN_{jit} + \sum_{k=14,j=2}^{k=29,j=17} \beta_k Year_{ji} + \sum_{k=30,j=2}^{k=45,j=16} \beta_k states_{jit} + u_i + v_i$
**M8**<br>znbin, BYM	$\eta_{it} = \alpha + \sum_{k=1,j=2}^{k=4,j=5} \beta_k GISDQ_{jit} + \beta_5 Sex_{it} + \sum_{k=6,j=2}^{k=9,j=5} \beta_k Age_{jit} + \sum_{k=10,j=2}^{k=13,j=5} \beta_k \%NonN_{jit} + \sum_{k=14,j=2}^{k=29,j=17} \beta_k Year_{ji} + \sum_{k=30,j=2}^{k=32,j=16} \beta_k GeoS_{jit} + u_i + v_i$
**M9**<br>znbin, BYM, non-parametric dynamic temporal trend	$\eta_{it} = \alpha + \sum_{k=1,j=2}^{k=4,j=5} \beta_k GISDQ_{jit} + \beta_5 Sex_{it} + \sum_{k=6,j=2}^{k=9,j=5} \beta_k Age_{jit} + \sum_{k=10,j=2}^{k=13,j=5} \beta_k \%NonN_{jit} + u_i + v_i + \gamma_t + \phi_t$
**M10**<br>znbin, BYM, parametric temporal trend	$\eta_{it} = \alpha + \sum_{k=1,j=2}^{k=4,j=5} \beta_k GISDQ_{jit} + \beta_5 Sex_{it} + \sum_{k=6,j=2}^{k=9,j=5} \beta_k Age_{jit} + \sum_{k=10,j=2}^{k=13,j=5} \beta_k \%NonN_{jit} + u_i + v_i + (\beta + \delta_i) \times t$

**M11**<br>znbin, BYM, non-parametric dynamic temporal trend	$\eta_{it} = \alpha + \sum_{k=1,j=2}^{k=4,j=5} \beta_k GISDQ_{jit} + \beta_5 Sex_{it} + \sum_{k=6,j=2}^{k=9,j=5} \beta_k Age_{jit} + \sum_{k=10,j=2}^{k=13,j=5} \beta_k \%NonN_{jit} + \sum_{k=14,j=2}^{k=29,j=16} \beta_k states_{jit} + u_i + v_i + \gamma_t + \phi_t$
**M12**<br>znbin, BYM, parametric temporal trend	$\eta_{it} = \alpha + \sum_{k=1,j=2}^{k=4,j=5} \beta_k GISDQ_{jit} + \beta_5 Sex_{it} + \sum_{k=6,j=2}^{k=9,j=5} \beta_k Age_{jit} + \sum_{k=10,j=2}^{k=13,j=5} \beta_k \%NonN_{jit} + \sum_{k=14,j=2}^{k=29,j=16} \beta_k states_{jit} + u_i + v_i + (\beta + \delta_i) \times t$
**M13**<br>znbin, BYM, non-parametric dynamic temporal trend	$\eta_{it} = \alpha + \sum_{k=1,j=2}^{k=4,j=5} \beta_k GISDQ_{jit} + \beta_5 Sex_{it} + \sum_{k=6,j=2}^{k=9,j=5} \beta_k Age_{jit} + \sum_{k=10,j=2}^{k=13,j=5} \beta_k \%NonN_{jit} + \sum_{k=14,j=2}^{k=16,j=4} \beta_k GeoS_{jit} + u_i + v_i + \gamma_t + \phi_t$
**M14**<br>znbin, BYM, parametric temporal trend	$\eta_{it} = \alpha + \sum_{k=1,j=2}^{k=4,j=5} \beta_k GISDQ_{jit} + \beta_5 Sex_{it} + \sum_{k=6,j=2}^{k=9,j=5} \beta_k Age_{jit} + \sum_{k=10,j=2}^{k=13,j=5} \beta_k \%NonN_{jit} + \sum_{k=14,j=2}^{k=16,j=4} \beta_k GeoS_{jit} + u_i + v_i + (\beta + \delta_i) \times t$

**Description of parameters and effects**
η_{it}: rate of measles in district i {1, …, 401} and time t {1, …, 17}
α: intercept
$\beta_k GISDQ_{jit}$: fixed-effects of socioeconomic deprivation quintiles j {2, …, 5} with quintile 5 (least deprived) as reference category in district i and time t
$\beta_5 Sex_{it}$: fixed-effect of sex in district i and time t
$\beta_k Age_{jit}$: fixed-effects of age groups 15-29, 30-44, 45-64 and 65+ with age group 0-14 as reference category in district i and time t.
$\beta_k \%NonN_{jit}$: fixed-effects of the quintiles of the proportion of non-nationals with quintile 1 (least proportion of non-nationals) in district i and time t.
$\beta_k Year_{ji}$: fixed-effects of time period {2002, …, 2017} with year 2001 as reference category in district i.
$\beta_k states_{ijt}$: fixed-effects of federal states {Berlin (BE), Baden-Württemberg (BW), Bavaria (BY), Bremen (HB), Hesse (HE), Hamburg (HH), Mecklenburg-Vorpommern (MV), Lower Saxony (NI), North Rhine-Westphalia (NW), Rhineland-Palatinate (RP), Schleswig-Holstein (SH), Saarland (SL), Saxony (SN), Saxony-Anhalt (ST), Thuringia (TH)} with Brandenburg (BB) as reference category in in district i and time t.
$\beta_k GeoS_{jit}$: fixed-effects of North-South-West-East geographic factors {eastern Germany, southern Germany, western Germany} with northern Germany as reference category in district I and time t.
$u_i + v_i$: structured and unstructured spatial random-effects (Besag-York-Mollié)
$\gamma_t + \phi_t$: structured and unstructured temporal random-effects (temporally non-parametric dynamic trend)
$(\beta + \delta_i) \times t$: main linear time trend and spatial differential time trend (temporally parametric trend)

Table S2: R syntax of the best fit model

```
Model_13_zinbin <-
        inla(measles_O ~ 1 + GISD_Q + sex + age + Per_Non_Nationals_ij_Q + North_South_East_West +
                        f(ID.area, model = "besag", graph = Ger.adj) +
                        f(ID.areaI, model = "iid") +
                        f(ID.year, model = "rw2") +
                        f(ID.yearI, model = "iid"),
                data = DATA, family = "zeroinflatednbinomial1", offset = log(measles_E),
                control.compute = list(waic = TRUE, smtp = "taucs"),
                control.predictor = list(compute = TRUE, precision = TRUE),
                control.inla = list(int.strategy = "eb", strategy = "gaussian"),
                num.threads = 10,
                verbose = TRUE)
```

Table S3: Summary statistic on measles incidence by sex, age-group and year

Year	Sex	Age 0-14	Age 15-29	Age 30-44	Age 45-64	Age 65+	Total
**2001**	**Male**	2373	742	91	13	2	3221
	**Female**	2136	654	126	29	6	2951
**2002**	**Male**	1978	307	56	11	1	2353
	**Female**	1940	302	107	15	2	2366
**2003**	**Male**	339	34	8	6	2	389
	**Female**	328	57	18	7	2	412
**2004**	**Male**	46	14	7	2	0	69
	**Female**	44	18	12	12	0	86
**2005**	**Male**	326	61	27	8	1	423
	**Female**	273	71	35	7	2	388
**2006**	**Male**	819	334	57	23	2	1235
	**Female**	691	300	111	21	8	1131
**2007**	**Male**	163	65	24	2	1	255
	**Female**	203	78	45	4	1	331
**2008**	**Male**	301	127	23	8	0	459
	**Female**	333	101	40	7	2	483
**2009**	**Male**	165	87	30	3	2	287
	**Female**	163	98	41	9	0	311
**2010**	**Male**	232	140	40	3	3	418
	**Female**	211	108	52	12	0	383
**2011**	**Male**	468	227	71	17	2	785
	**Female**	489	230	111	17	4	851
**2012**	**Male**	38	26	15	5	2	86
	**Female**	32	37	27	3	1	100
**2013**	**Male**	408	335	137	26	1	907
	**Female**	421	263	183	60	1	928
**2014**	**Male**	106	96	51	13	0	266
	**Female**	115	70	47	10	2	244
**2015**	**Male**	682	439	217	69	9	1416
	**Female**	558	348	209	63	3	1181
**2016**	**Male**	111	54	28	8	0	201
	**Female**	88	41	14	14	2	159
**2017**	**Male**	244	152	80	30	4	510
	**Female**	247	135	97	41	4	524
**Total**		17071	6151	2237	578	72	26109

Table S4: Results of the models M0 to M6 – RR (95%-CrI)

Fixed Predictor variables		M0_P	M0_znbin	M1	M2	M3	M4	M5	M6
**Intercept**	**(Intercept)**	401·12 (396·3-405·98)	360·24 (348·15-372·61)	NA	179·11 (154·1-206·94)	175·43 (150·12 - 203·71)	158·39 (135·27 - 184·26)	77·65 (62·76-94·96)	310·6 (247·93-384·1)
**Area Deprivation (GISD)**	**Q1**				1·39 (1·06-1·79)	1·4 (1·07-1·8)	1·38 (1·06-1·78)	1·3 (1-1·67)	1·62 (1·26-2·06)
	**Q2**				1·1 (0·88-1·36)	1·1 (0·88-1·37)	1·1 (0·87-1·36)	1·03 (0·82-1·27)	1·25 (1·01-1·54)
	**Q3**				1·05 (0·86-1·26)	1·05 (0·86-1·27)	1·04 (0·86-1·26)	0·99 (0·82-1·19)	1·06 (0·88-1·26)
	**Q4**				1·23 (1·04-1·44)	1·23 (1·04-1·44)	1·23 (1·04-1·43)	1·2 (1·02-1·4)	1·23 (1·05-1·43)
**Sex**	**Female**					1·04 (0·97-1·1)	1·03 (0·97-1·1)	1·04 (0·97-1·11)	1·05 (0·99-1·11)
**Age groups**	**15-29**						1·24 (1·15-1·34)	1 (0·91-·09)	1·05 (0·96-1·14)
	**30-44**						1·13 (1·03-1·23)	0·9 (0·81-1)	1·04 (0·94-1·15)
	**45-64**						1·22 (1·07-1·37)	1·21 (1·07-1·36)	1·34 (1·19-1·51)
	**65+**						1·27 (0·97-1·63)	1·75 (1·33-2·27)	1·82 (1·38-2·36)
**%Non-Nationals**	**%NNQ2**							1·86 (1·57-2·19)	1·78 (1·51-2·09)
	**%NNQ3**							2·38 (1·98-2·83)	2·25 (1·87-2·68)
	**%NNQ4**							2·75 (2·25-3·33)	2·4 (1·95-2·92)
	**%NNQ5**							3·52 (2·78-4·38)	3·02 (2·36-3·8)
**Years**	**2002**								0·58 (0·51-0·67)
	**2003**								0·13 (0·11-0·15)
	**2004**								0·03 (0·03-0·04)
	**2005**								0·13 (0·11-0·15)
	**2006**								0·34 (0·29-0·4)
	**2007**								0·1 (0·08-0·12)

	**2008**								0·2 (0·17-0·23)
	**2009**								0·1 (0·08-0·11)
	**2010**								0·15 (0·13-0·18)
	**2011**								0·32 (0·27-0·37)
	**2012**								0·05 (0·04-0·06)
	**2013**								0·3 (0·25-0·34)
	**2014**								0·1 (0·08-0·12)
	**2015**								0·34 (0·29-0·4)
	**2016**								0·07 (0·05-0·08)
	**2017**								0·18 (0·15-0·21)
**Geographic factors**	**Eastern Germany**								
	**Southern Germany**								
	**Western Germany**								
**Model fit**	**WAIC**	140732	56221	NA	52913	52915	52895	52791	50113
	**Effective number of paramters**	59.01653	5.0858726	NA	350.8560	353.129	357.8625	358.7994	380.9468
**Hyperparameter and random-effects**	**size for nbinomial zero-inflated observation**		0.0042034	NA	0.005111	0.004837	0.004881	0.004679	0.0032726
	**zero-probability parameter for zero-inflated nbinomial**		0.0986697	NA	0.161675	0.161455	0.161826	0.164111	0.2384291
	**Precision for Besags ICAR model (area)**				0.52782	0.559772	0.493952	0.534346	0.647542
	**Precision for IID model (area)**				0.52773	0.557464	0.492448	0.533593	0.644793
	**Precision for rw2 model (year)**								
	**Precision for IID model (year)**								

Table S5: Results of the models M7 to M14 – RR (95%-CrI)

Fixed Predictor variables		M7	M8	M9	M10	M11	M12	M13	M14
**Intercept**	**(Intercept)**	519·12 (195·36-1125·13)	332·99 (192·27-537·49)	52·29 (33·83-77·22)	NA	87·24 (29·64-201·23)	NA	58·67 (28·57-107·24)	NA
**Area Deprivation (GISD)**	**Q1**	1·63 (1·26-2·08)	1·62 (1·25-2·05)	1·61 (1·25-2·04)	NA	1·62 (1·25-2·06)	NA	1·58 (1·23-2)	NA
	**Q2**	1·24 (1-1·53)	1·25 (1·01-1·54)	1·24 (1-1·53)	NA	1·24 (0·99-1·53)	NA	1·23 (0·99-1·51)	NA
	**Q3**	1·04 (0·86-1·24)	1·06 (0·88-1·27)	1·05 (0·88-1·26)	NA	1·04 (0·86-1·24)	NA	1·05 (0·87-1·26)	NA
	**Q4**	1·2 (1·03-1·4)	1·24 (1·06-1·43)	1·23 (1·05-1·43)	NA	1·2 (1·03-1·4)	NA	1·23 (1·05-1·43)	NA
**Sex**	**Female**	1·05 (0·99-1·11)	1·05 (0·99-1·11)	1·05 (0·99-1·11)	NA	1·05 (0·99-1·11)	NA	1·05 (0·99-1·11)	NA
**Age groups**	**15-29**	1·05 (0·96-1·15)	1·05 (0·96-1·14)	1·05 (0·96-1·14)	NA	1·05 (0·96-1·15)	NA	1·05 (0·96-1·14)	NA
	**30-44**	1·04 (0·94-1·15)	1·04 (0·94-1·15)	1·04 (0·94-1·15)	NA	1·04 (0·94-1·15)	NA	1·04 (0·94-1·15)	NA
	**45-64**	1·34 (1·18-1·51)	1·34 (1·19-1·51)	1·34 (1·19-1·51)	NA	1·34 (1·18-1·51)	NA	1·34 (1·19-1·51)	NA
	**65+**	1·8 (1·36-2·34)	1·83 (1·38-2·36)	1·83 (1·39-2·37)	NA	1·81 (1·37-2·34)	NA	1·83 (1·38-2·37)	NA
**%Non-Nationals**	**non_natQ2**	1·76 (1·49-2·07)	1·79 (1·51-2·1)	1·78 (1·51-2·09)	NA	1·76 (1·49-2·07)	NA	1·78 (1·5-2·09)	NA
	**non_natQ3**	2·22 (1·85-2·66)	2·26 (1·88-2·69)	2·25 (1·87-2·68)	NA	2·22 (1·85-2·65)	NA	2·24 (1·87-2·67)	NA
	**non_natQ4**	2·36 (1·91-2·88)	2·41 (1·96-2·93)	2·4 (1·95-2·92)	NA	2·36 (1·91-2·88)	NA	2·39 (1·94-2·91)	NA
	**non_natQ5**	2·96 (2·3-3·74)	3·03 (2·37-3·82)	3·02 (2·36-3·8)	NA	2·95 (2·3-3·73)	NA	3·01 (2·35-3·8)	NA
**Years**	**2002**	0·58 (0·5-0·67)	0·58 (0·51-0·67)						
	**2003**	0·13 (0·11-0·15)	0·13 (0·11-0·15)						
	**2004**	0·03 (0·03-0·04)	0·03 (0·03-0·04)						
	**2005**	0·13 (0·11-0·15)	0·13 (0·11-0·15)						
	**2006**	0·34 (0·29-0·39)	0·34 (0·29-0·4)						
	**2007**	0·1 (0·08-0·12)	0·1 (0·08-0·12)						
	**2008**	0·2 (0·17-0·23)	0·2 (0·17-0·23)						
	**2009**	0·09 (0·08-0·11)	0·1 (0·08-0·11)						
	**2010**	0·15 (0·13-0·18)	0·15 (0·13-0·18)						
	**2011**	0·32	0·32						

		(0·27-0·37)	(0·27-0·37)						
	**2012**	0·05 (0·04-0·06)	0·05 (0·04-0·06)						
	**2013**	0·3 (0·25-0·34)	0·3 (0·25-0·34)						
	**2014**	0·1 (0·08-0·12)	0·1 (0·08-0·12)						
	**2015**	0·34 (0·29-0·4)	0·34 (0·29-0·4)						
	**2016**	0·07 (0·05-0·08)	0·07 (0·05-0·08)						
	**2017**	0·18 (0·15-0·21)	0·17 (0·15-0·21)						
	**Jahr**				NA		NA		NA
**Federal States**	**BE**	4·05 (0·89-11·77)				3·8 (0·9-10·66)			
	**BW**	0·72 (0·18-1·93)				0·74 (0·19-2)			
	**BY**	1·05 (0·29-2·72)				1·06 (0·29-2·78)			
	**HB**	0·89 (0·14-3·05)				0·91 (0·14-3·1)			
	**HE**	0·65 (0·19-1·65)				0·65 (0·18-1·66)			
	**HH**	1·99 (0·22-7·7)				1·96 (0·22-7·54)			
	**MV**	0·3 (0·09-0·77)				0·31 (0·09-0·79)			
	**NI**	0·92 (0·32-2·1)				0·94 (0·32-2·18)			
	**NW**	0·58 (0·17-1·47)				0·58 (0·16-1·49)			
	**RP**	0·67 (0·18-1·77)				0·67 (0·18-1·79)			
	**SH**	1·2 (0·22-3·78)				1·21 (0·22-3·86)			
	**SL**	0·27 (0·04-0·94)				0·27 (0·04-0·96)			
	**SN**	0·75 (0·26-1·69)				0·75 (0·26-1·71)			
	**ST**	0·71 (0·28-1·5)				0·73 (0·28-1·54)			
	**TH**	0·67 (0·21-1·59)				0·65 (0·21-1·56)			
**Geographic factors**	**Eastern Germany**		1·04 (0·53-1·84)				NA	0·96 (0·47-1·73)	NA
	**Southern Germany**		1·22 (0·53-2·42)				NA	1·22 (0·51-2·46)	NA
	**Western Germany**		0·79 (0·41-1·37)				NA	0·75 (0·39-1·32)	NA
**Model fit**	**WAIC**	50116.330	50114.228	50114.168	NA	50116.708	NA	50113.355	NA
	**Effective**	384.48392	381.71258	378.83482	NA	382.41124	NA	375.35656	NA

	**number of paramters**								
**Hyperparameter and random-effects**	**size for nbinomial zero-inflated observations**	0.0033772	0.0032301	0.0033896	NA	0.0034236	NA	0.0029260	NA
	**zero-probability parameter for zero-inflated nbinomial**	0.2386235	0.238878	0.2384780	NA	0.2386762	NA	0.2382197	NA
	**Precision for Besags ICAR model (area)**	0.7394203	0.5826114	0.591476	NA	0.6781552	NA	0.44574	NA
	**Precision for IID model (area)**	0.7343922	0.5832253	65556.85	NA	18541.82	NA	17367.2	NA
	**Precision for rw2 model (year)**				NA	0.672951	NA	0.4448313	NA
	**Precision for IID model (year)**				NA	67972.69	NA	61514.93	NA

Temporal random-effects

The posterior temporal trends of the structured (rw2 model) and unstructured (iid model) temporal random-effects were calculated using the marginal of the random-effects (Figure S.1). The structured effect shows a decreasing trend, while visible fluctuations are present for the unstructured effect. By combining the marginals of the structured and unstructured temporal random-effects (temporal main effect, TME), we computed relative risks of the posterior effects with corresponding 95% credible intervals (95%-CrI) (Figure S.2). The temporal risk of measles infections corresponds to the fluctuations of the unstructured effect, hence the structured effect has less meaningful impact.

Figure S1: Posterior temporal trend of measles infections in Germany

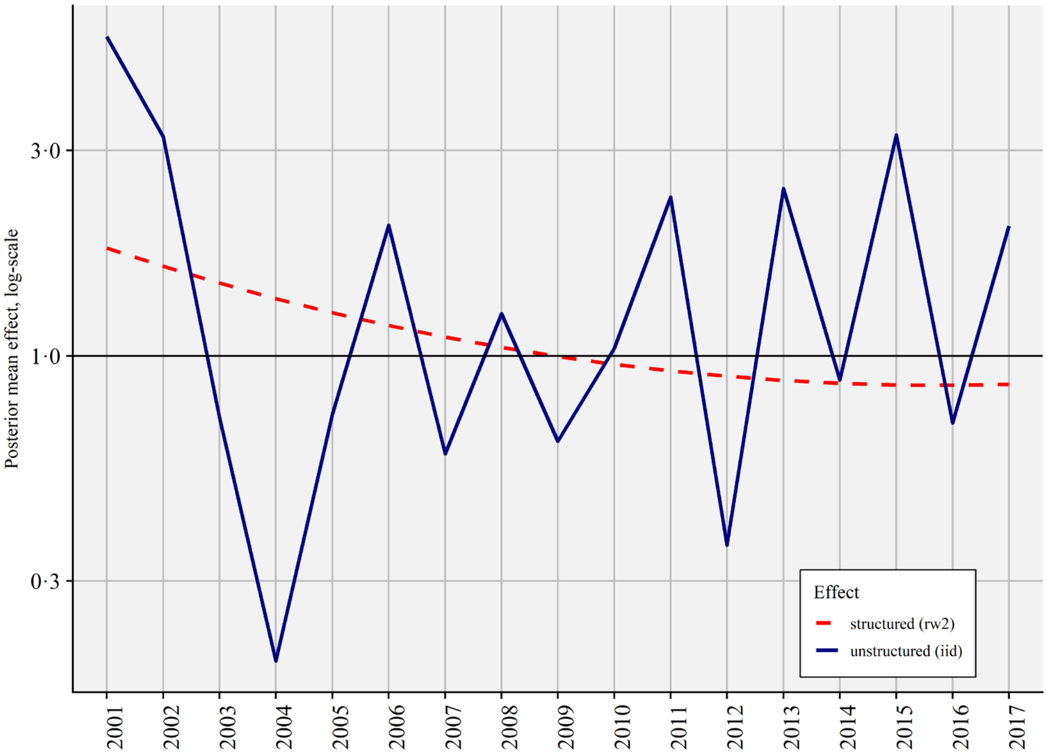

Figure S2: Posterior temporal relative risks of measles infections in Germany

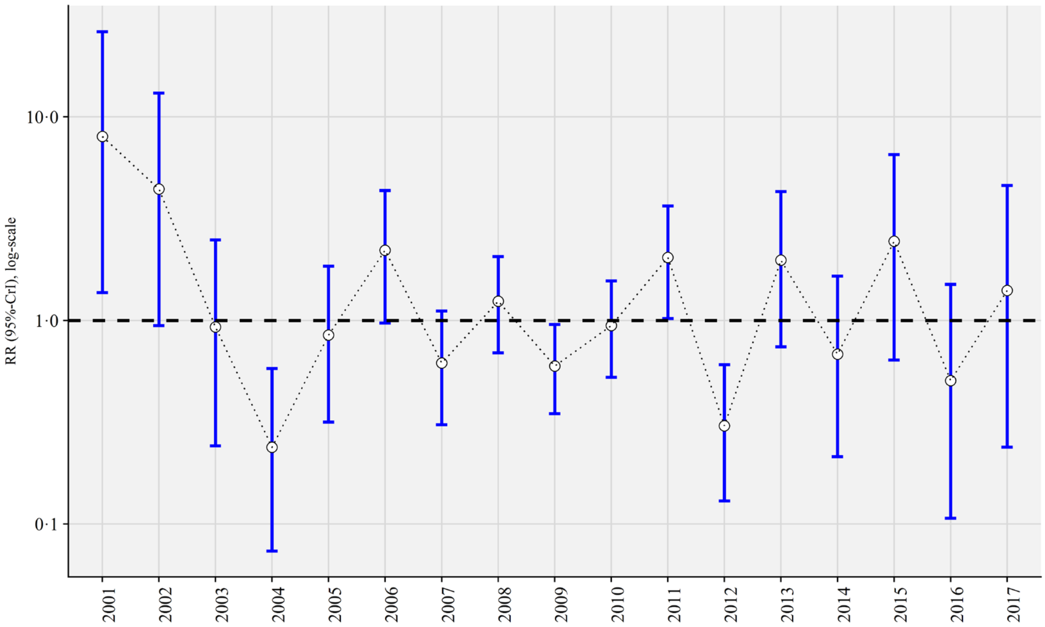

Figure S3: Reference map of the federal states in Germany

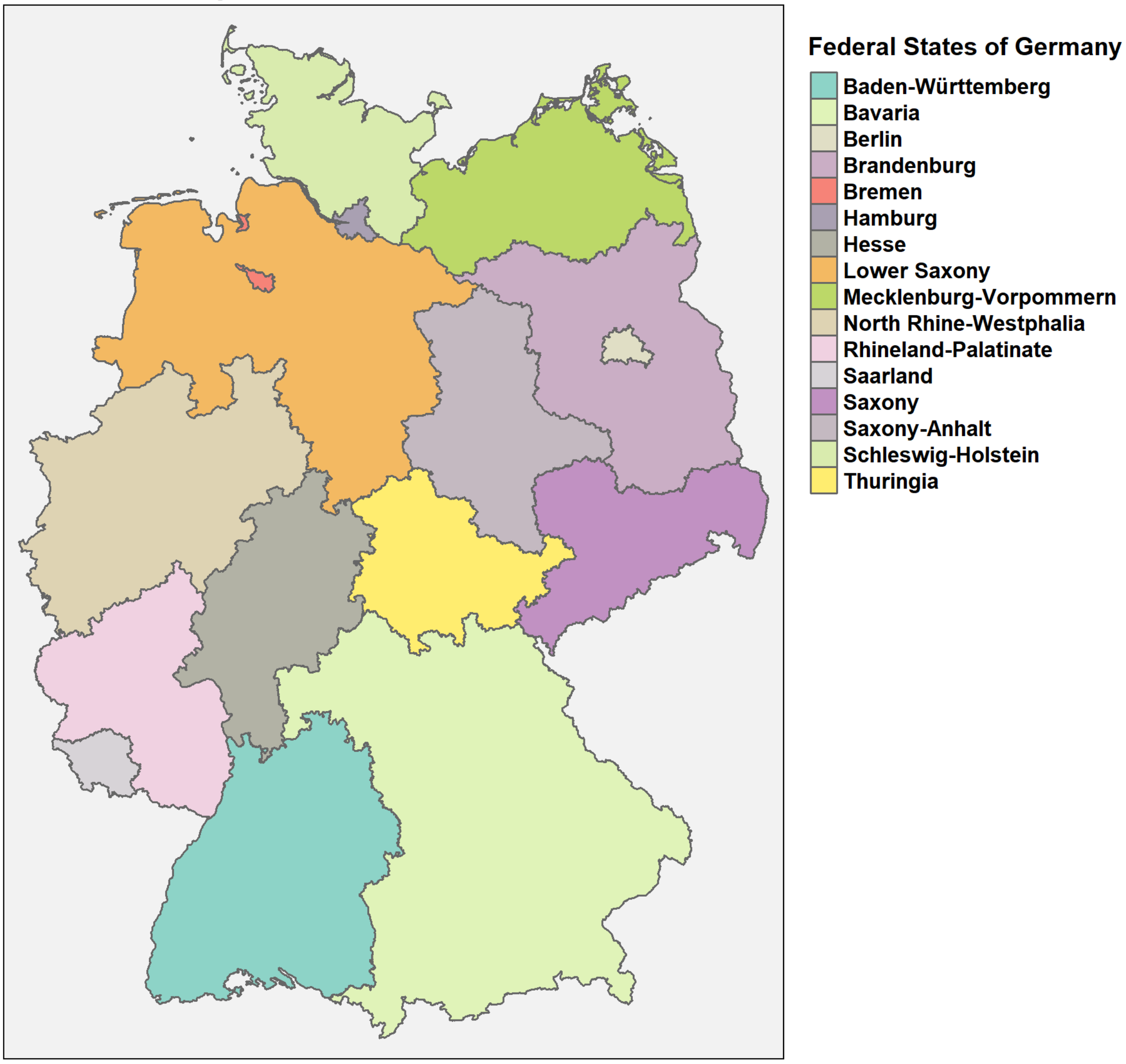

Figure S4: Bayesian regression adjusted spatial relative risk (ARR) and exceedance thresholds by German federals states

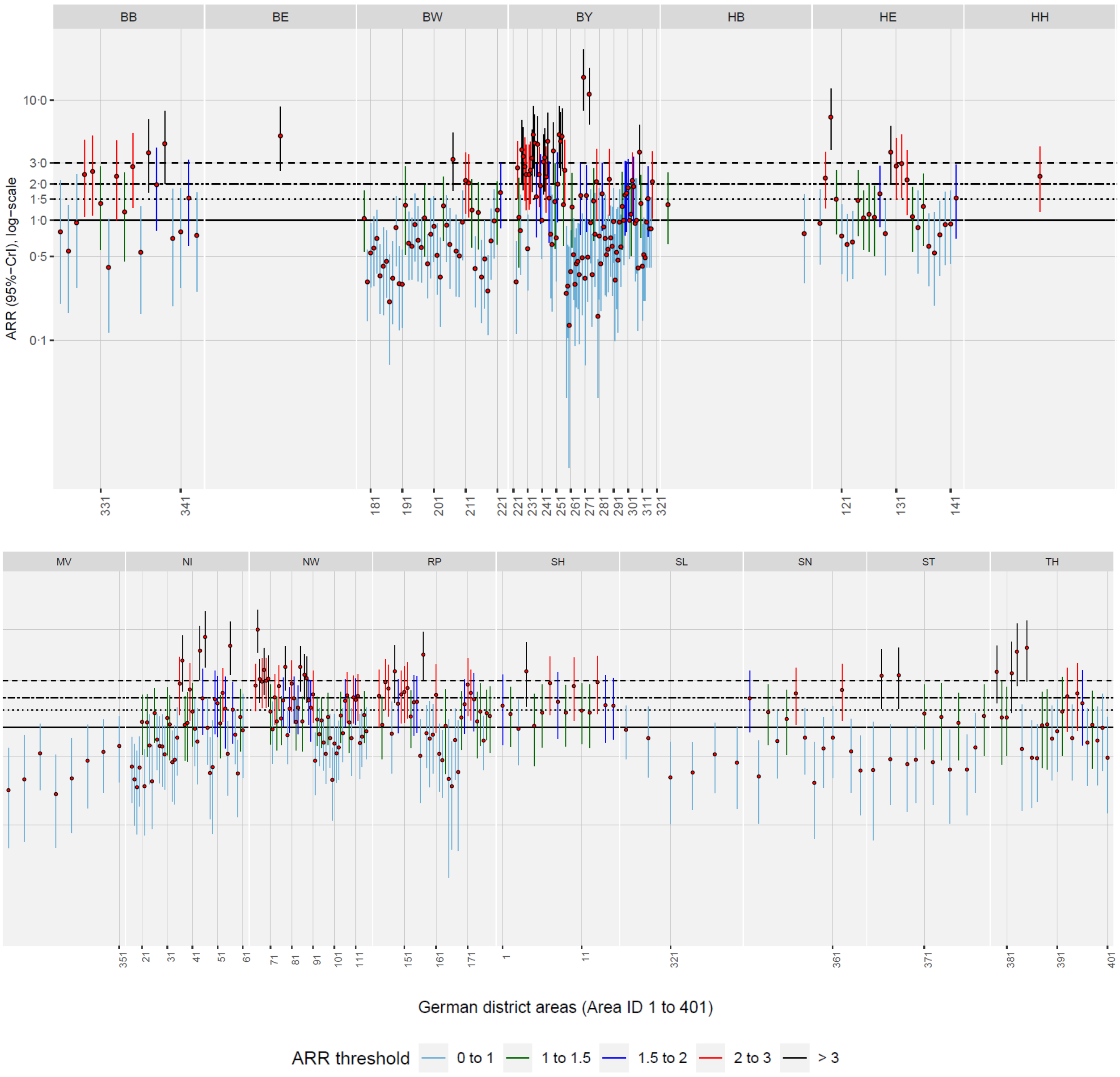

Note: BB (Brandenburg), BE (Berlin), BW (Baden-Württemberg), BY (Bavaria), HB (Bremen), HE (Hesse), HH (Hamburg), MV (Mecklenburg-Vorpommern), NI (Lower Saxony), NW (North Rhine-Westphalia), RP (Rhineland Palatinate), SH (Schleswig-Holstein), SL (Saarland), SN (Saxony), ST (Saxony-Anhalt), TH (Thuringia)

Table S6: District-specific adjusted relative risk

District	Area ID	ARR (95% credible interval)
Flensburg, Kreisfreie Stadt	1	1·65 (0·66-3·46)
Kiel, Landeshauptstadt, Kreisfreie Stadt	2	1·36 (0·63-2·57)
Lübeck, Hansestadt, Kreisfreie Stadt	3	0·96 (0·41-1·91)
Neumünster, Kreisfreie Stadt	4	3·73 (1·63-7·36)
Dithmarschen, Landkreis	5	1·04 (0·4-2·21)
Herzogtum Lauenburg, Landkreis	6	1·41 (0·63-2·74)
Nordfriesland, Landkreis	7	2·83 (1·34-5·29)
Ostholstein, Landkreis	8	1·81 (0·77-3·64)
Pinneberg, Landkreis	9	1·4 (0·64-2·69)
Plön, Landkreis	10	2·65 (1·17-5·17)
Rendsburg-Eckernförde, Landkreis	11	1·48 (0·68-2·81)
Schleswig-Flensburg, Landkreis	12	1·4 (0·62-2·75)
Segeberg, Landkreis	13	2·87 (1·37-5·34)
Steinburg, Landkreis	14	1·68 (0·7-3·41)
Stormarn, Landkreis	15	1·65 (0·75-3·16)
Hamburg	16	2·32 (1·18-4·1)
Braunschweig, Kreisfreie Stadt	17	0·4 (0·17-0·79)
Salzgitter, Kreisfreie Stadt	18	0·29 (0·1-0·68)
Wolfsburg, Kreisfreie Stadt	19	0·24 (0·08-0·57)
Gifhorn, Landkreis	20	0·39 (0·15-0·81)
Goslar, Landkreis	21	1·13 (0·52-2·15)
Helmstedt, Landkreis	22	0·25 (0·08-0·59)
Northeim, Landkreis	23	1·12 (0·5-2·16)
Peine, Landkreis	24	0·65 (0·26-1·35)
Wolfenbüttel, Landkreis	25	0·28 (0·09-0·66)
Göttingen, Landkreis	26	1·39 (0·7-2·49)
Region Hannover, Landkreis	27	0·75 (0·37-1·36)
Diepholz, Landkreis	28	0·64 (0·27-1·3)
Hameln-Pyrmont, Landkreis	29	0·63 (0·25-1·34)
Hildesheim, Landkreis	30	0·52 (0·23-1·03)
Holzminden, Landkreis	31	1·25 (0·52-2·54)
Nienburg (Weser), Landkreis	32	1·07 (0·46-2·12)
Schaumburg, Landkreis	33	0·44 (0·17-0·92)
Celle, Landkreis	34	0·46 (0·16-1·05)
Cuxhaven, Landkreis	35	0·78 (0·32-1·59)
Harburg, Landkreis	36	2·8 (1·33-5·2)
Lüchow-Dannenberg, Landkreis	37	4·78 (2·33-8·74)
Lüneburg, Landkreis	38	1·05 (0·46-2·08)
Osterholz, Landkreis	39	1·1 (0·45-2·28)
Rotenburg (Wümme), Landkreis	40	2·44 (1·16-4·52)
Heidekreis, Landkreis	41	1·45 (0·64-2·85)
Stade, Landkreis	42	0·96 (0·4-1·95)
Uelzen, Landkreis	43	0·71 (0·26-1·56)

Verden, Landkreis	44	6·05 (3·02-10·86)
Delmenhorst, Kreisfreie Stadt	45	1·98 (0·84-3·97)
Emden, Kreisfreie Stadt	46	8·4 (4·09-15·37)
Oldenburg (Oldenburg), Kreisfreie Stadt	47	0·99 (0·41-2)
Osnabrück, Kreisfreie Stadt	48	0·34 (0·12-0·79)
Wilhelmshaven, Kreisfreie Stadt	49	0·39 (0·08-1·17)
Ammerland, Landkreis	50	1·93 (0·85-3·79)
Aurich, Landkreis	51	1·75 (0·8-3·35)
Cloppenburg, Landkreis	52	1·09 (0·48-2·13)
Emsland, Landkreis	53	2·25 (1·13-4·02)
Friesland, Landkreis	54	1·55 (0·61-3·28)
Grafschaft Bentheim, Landkreis	55	0·53 (0·2-1·15)
Leer, Landkreis	56	6·76 (3·44-11·98)
Oldenburg, Landkreis	57	1·51 (0·69-2·91)
Osnabrück, Landkreis	58	0·84 (0·42-1·49)
Vechta, Landkreis	59	0·33 (0·12-0·74)
Wesermarsch, Landkreis	60	1·27 (0·57-2·47)
Wittmund, Landkreis	61	0·93 (0·3-2·2)
Bremen, Kreisfreie Stadt	62	1·34 (0·64-2·48)
Bremerhaven, Kreisfreie Stadt	63	0·77 (0·3-1·65)
Düsseldorf, Kreisfreie Stadt	64	2·65 (1·47-4·42)
Duisburg, Kreisfreie Stadt	65	9·9 (5·77-15·86)
Essen, Kreisfreie Stadt	66	3·09 (1·77-5)
Krefeld, Kreisfreie Stadt	67	2·94 (1·57-5·03)
Mönchengladbach, Kreisfreie Stadt	68	3·86 (2·14-6·43)
Mülheim an der Ruhr, Kreisfreie Stadt	69	2·98 (1·58-5·12)
Oberhausen, Kreisfreie Stadt	70	3·11 (1·63-5·41)
Remscheid, Kreisfreie Stadt	71	1·44 (0·67-2·74)
Solingen, Kreisfreie Stadt	72	0·95 (0·43-1·83)
Wuppertal, Kreisfreie Stadt	73	2·02 (1·05-3·5)
Kleve, Kreis	74	1·15 (0·58-2·05)
Mettmann, Kreis	75	2·62 (1·49-4·29)
Rhein-Kreis Neuss	76	1·22 (0·66-2·06)
Viersen, Kreis	77	1·89 (1·02-3·2)
Wesel, Kreis	78	4·14 (2·37-6·71)
Bonn, Kreisfreie Stadt	79	0·82 (0·42-1·46)
Köln, Kreisfreie Stadt	80	1·55 (0·88-2·54)
Leverkusen, Kreisfreie Stadt	81	2·78 (1·44-4·84)
Städteregion Aachen (einschl. Stadt Aachen)	82	1·98 (1·1-3·3)
Düren, Kreis	83	1·13 (0·58-2)
Rhein-Erft-Kreis	84	1·57 (0·85-2·66)
Euskirchen, Kreis	85	4·11 (2·32-6·75)
Heinsberg, Kreis	86	1·14 (0·57-2·06)
Oberbergischer Kreis	87	3·42 (1·94-5·58)
Rheinisch-Bergischer Kreis	88	3·18 (1·77-5·26)

Rhein-Sieg-Kreis	89	1·88 (1·06-3·08)
Bottrop, Kreisfreie Stadt	90	1·58 (0·72-3·04)
Gelsenkirchen, Kreisfreie Stadt	91	2·15 (1·11-3·77)
Münster, Kreisfreie Stadt	92	0·45 (0·2-0·88)
Borken, Kreis	93	1·2 (0·64-2·05)
Coesfeld, Kreis	94	0·84 (0·4-1·57)
Recklinghausen, Kreis	95	1·17 (0·61-2·01)
Steinfurt, Kreis	96	0·7 (0·36-1·22)
Warendorf, Kreis	97	0·53 (0·26-0·97)
Bielefeld, Kreisfreie Stadt	98	1·28 (0·66-2·24)
Gütersloh, Kreis	99	0·77 (0·39-1·37)
Herford, Kreis	100	0·29 (0·11-0·62)
Höxter, Kreis	101	0·68 (0·29-1·36)
Lippe, Kreis	102	0·54 (0·27-0·96)
Minden-Lübbecke, Kreis	103	0·62 (0·29-1·16)
Paderborn, Kreis	104	1·32 (0·69-2·29)
Bochum, Kreisfreie Stadt	105	0·87 (0·44-1·54)
Dortmund, Kreisfreie Stadt	106	1·85 (1·01-3·11)
Hagen, Kreisfreie Stadt	107	2·11 (1·11-3·65)
Hamm, Kreisfreie Stadt	108	1·12 (0·55-2·03)
Herne, Kreisfreie Stadt	109	0·77 (0·32-1·57)
Ennepe-Ruhr-Kreis	110	2·01 (1·09-3·39)
Hochsauerlandkreis	111	1·91 (1·06-3·16)
Märkischer Kreis	112	2·1 (1·16-3·49)
Olpe, Kreis	113	0·69 (0·3-1·37)
Siegen-Wittgenstein, Kreis	114	0·8 (0·39-1·45)
Soest, Kreis	115	1·33 (0·69-2·31)
Unna, Kreis	116	0·91 (0·47-1·59)
Darmstadt, Kreisfreie Stadt	117	0·94 (0·43-1·79)
Frankfurt am Main, Kreisfreie Stadt	118	2·25 (1·26-3·7)
Offenbach am Main, Kreisfreie Stadt	119	7·23 (3·85-12·41)
Wiesbaden, Landeshauptstadt, Kreisfreie Stadt	120	1·49 (0·77-2·62)
Bergstraße, Landkreis	121	0·73 (0·36-1·34)
Darmstadt-Dieburg, Landkreis	122	0·63 (0·31-1·13)
Groß-Gerau, Landkreis	123	0·65 (0·32-1·18)
Hochtaunuskreis	124	1·46 (0·74-2·61)
Main-Kinzig-Kreis	125	1·05 (0·57-1·77)
Main-Taunus-Kreis	126	1·12 (0·55-2·03)
Odenwaldkreis	127	1·05 (0·5-1·94)
Offenbach, Landkreis	128	1·65 (0·88-2·83)
Rheingau-Taunus-Kreis	129	0·77 (0·35-1·47)
Wetteraukreis	130	3·68 (2·06-6·08)
Gießen, Landkreis	131	2·81 (1·48-4·84)
Lahn-Dill-Kreis	132	2·95 (1·54-5·14)
Limburg-Weilburg, Landkreis	133	2·17 (1·11-3·84)
Marburg-Biedenkopf, Landkreis	134	1·07 (0·55-1·89)

Vogelsbergkreis	135	0·87 (0·36-1·77)
Kassel, Kreisfreie Stadt	136	1·31 (0·61-2·45)
Fulda, Landkreis	137	0·6 (0·28-1·14)
Hersfeld-Rotenburg, Landkreis	138	0·53 (0·2-1·17)
Kassel, Landkreis	139	0·75 (0·34-1·44)
Schwalm-Eder-Kreis	140	0·91 (0·42-1·73)
Waldeck-Frankenberg, Landkreis	141	0·93 (0·43-1·76)
Werra-Meißner-Kreis	142	1·53 (0·7-2·9)
Koblenz, Kreisfreie Stadt	143	2·08 (1-3·86)
Ahrweiler, Landkreis	144	1·05 (0·47-2·04)
Altenkirchen (Westerwald), Landkreis	145	2·88 (1·5-5·02)
Bad Kreuznach, Landkreis	146	2·49 (1·29-4·37)
Birkenfeld, Landkreis	147	0·86 (0·34-1·81)
Cochem-Zell, Landkreis	148	3·72 (1·79-6·88)
Mayen-Koblenz, Landkreis	149	1·73 (0·87-3·09)
Neuwied, Landkreis	150	2·15 (1·1-3·8)
Rhein-Hunsrück-Kreis	151	2·29 (1·1-4·21)
Rhein-Lahn-Kreis	152	2·52 (1·23-4·59)
Westerwaldkreis	153	1·29 (0·62-2·38)
Trier, Kreisfreie Stadt	154	1·79 (0·83-3·41)
Bernkastel-Wittlich, Landkreis	155	1·81 (0·88-3·31)
Eifelkreis Bitburg-Prüm	156	0·51 (0·17-1·2)
Vulkaneifel, Landkreis	157	5·52 (2·95-9·43)
Trier-Saarburg, Landkreis	158	0·87 (0·38-1·69)
Frankenthal (Pfalz), Kreisfreie Stadt	159	0·77 (0·24-1·84)
Kaiserslautern, Kreisfreie Stadt	160	0·83 (0·34-1·72)
Landau in der Pfalz, Kreisfreie Stadt	161	2·14 (0·88-4·41)
Ludwigshafen am Rhein, Kreisfreie Stadt	162	0·53 (0·22-1·09)
Mainz, Kreisfreie Stadt	163	0·46 (0·19-0·93)
Neustadt an der Weinstraße, Kreisfreie Stadt	164	1·03 (0·33-2·44)
Pirmasens, Kreisfreie Stadt	165	0·3 (0·03-1·2)
Speyer, Kreisfreie Stadt	166	0·25 (0·06-0·72)
Worms, Kreisfreie Stadt	167	0·74 (0·29-1·56)
Zweibrücken, Kreisfreie Stadt	168	0·35 (0·05-1·18)
Alzey-Worms, Landkreis	169	1·25 (0·59-2·34)
Bad Dürkheim, Landkreis	170	1·72 (0·86-3·08)
Donnersbergkreis	171	2·73 (1·3-5·07)
Germersheim, Landkreis	172	2·24 (1·17-3·89)
Kaiserslautern, Landkreis	173	1·92 (0·92-3·57)
Kusel, Landkreis	174	1·14 (0·48-2·3)
Südliche Weinstraße, Landkreis	175	1·44 (0·64-2·81)
Rhein-Pfalz-Kreis	176	0·73 (0·37-1·31)
Mainz-Bingen, Landkreis	177	1·4 (0·74-2·4)
Südwestpfalz, Landkreis	178	1·3 (0·55-2·61)
Stuttgart, Landeshauptstadt, Stadtkreis	179	1·03 (0·55-1·77)

Böblingen, Landkreis	180	0·3 (0·14-0·57)
Esslingen, Landkreis	181	0·54 (0·28-0·94)
Göppingen, Landkreis	182	0·58 (0·28-1·07)
Ludwigsburg, Landkreis	183	0·7 (0·36-1·22)
Rems-Murr-Kreis, Landkreis	184	0·34 (0·17-0·63)
Heilbronn, Stadtkreis	185	0·41 (0·16-0·87)
Heilbronn, Landkreis	186	0·45 (0·23-0·82)
Hohenlohekreis, Landkreis	187	0·21 (0·06-0·52)
Schwäbisch Hall, Landkreis	188	0·33 (0·14-0·67)
Main-Tauber-Kreis, Landkreis	189	0·86 (0·42-1·59)
Heidenheim, Landkreis	190	0·3 (0·12-0·61)
Ostalbkreis, Landkreis	191	0·29 (0·13-0·57)
Baden-Baden, Stadtkreis	192	1·32 (0·52-2·79)
Karlsruhe, Stadtkreis	193	0·64 (0·32-1·17)
Karlsruhe, Landkreis	194	0·61 (0·32-1·05)
Rastatt, Landkreis	195	0·92 (0·45-1·66)
Heidelberg, Stadtkreis	196	0·68 (0·31-1·31)
Mannheim, Stadtkreis	197	0·59 (0·3-1·05)
Neckar-Odenwald-Kreis, Landkreis	198	1·05 (0·5-1·94)
Rhein-Neckar-Kreis, Landkreis	199	0·44 (0·22-0·76)
Pforzheim, Stadtkreis	200	0·76 (0·33-1·52)
Calw, Landkreis	201	0·89 (0·45-1·58)
Enzkreis, Landkreis	202	0·51 (0·24-0·94)
Freudenstadt, Landkreis	203	0·34 (0·13-0·72)
Freiburg im Breisgau, Stadtkreis	204	1·31 (0·67-2·31)
Breisgau-Hochschwarzwald, Landkreis	205	0·9 (0·45-1·62)
Emmendingen, Landkreis	206	0·63 (0·27-1·25)
Ortenaukreis, Landkreis	207	3·2 (1·77-5·34)
Rottweil, Landkreis	208	0·55 (0·23-1·11)
Schwarzwald-Baar-Kreis, Landkreis	209	0·5 (0·23-0·95)
Tuttlingen, Landkreis	210	0·96 (0·46-1·75)
Konstanz, Landkreis	211	2·13 (1·14-3·64)
Lörrach, Landkreis	212	2·03 (1·05-3·55)
Waldshut, Landkreis	213	1·21 (0·6-2·2)
Reutlingen, Landkreis	214	0·4 (0·19-0·74)
Tübingen, Landkreis	215	1·15 (0·58-2·07)
Zollernalbkreis, Landkreis	216	0·34 (0·14-0·68)
Ulm, Stadtkreis	217	0·47 (0·19-1)
Alb-Donau-Kreis, Landkreis	218	0·26 (0·11-0·51)
Biberach, Landkreis	219	0·67 (0·32-1·25)
Bodenseekreis, Landkreis	220	0·98 (0·48-1·8)
Ravensburg, Landkreis	221	1·21 (0·63-2·1)
Sigmaringen, Landkreis	222	1·69 (0·86-3)
Ingolstadt	223	0·31 (0·11-0·67)
München, Landeshauptstadt	224	2·73 (1·5-4·56)
Rosenheim	225	1·05 (0·41-2·24)

Altötting, Landkreis	226	0·82 (0·35-1·62)
Berchtesgadener Land, Landkreis	227	3·87 (1·99-6·82)
Bad Tölz-Wolfratshausen, Landkreis	228	3·41 (1·79-5·91)
Dachau, Landkreis	229	2·8 (1·47-4·85)
Ebersberg, Landkreis	230	2·4 (1·22-4·25)
Eichstätt, Landkreis	231	0·58 (0·26-1·11)
Erding, Landkreis	232	2·4 (1·23-4·23)
Freising, Landkreis	233	2·59 (1·36-4·49)
Fürstenfeldbruck, Landkreis	234	3·27 (1·73-5·64)
Garmisch-Partenkirchen, Landkreis	235	5·15 (2·71-8·91)
Landsberg am Lech, Landkreis	236	4·37 (2·31-7·53)
Miesbach, Landkreis	237	1·56 (0·73-2·93)
Mühldorf a.Inn, Landkreis	238	4·3 (2·3-7·35)
München, Landkreis	239	2·4 (1·29-4·07)
Neuburg-Schrobenhausen, Landkreis	240	1·93 (0·94-3·54)
Pfaffenhofen a.d.Ilm, Landkreis	241	1 (0·48-1·85)
Rosenheim, Landkreis	242	3·05 (1·65-5·16)
Starnberg, Landkreis	243	3·33 (1·74-5·8)
Traunstein, Landkreis	244	2·3 (1·16-4·09)
Weilheim-Schongau, Landkreis	245	4·55 (2·41-7·82)
Landshut	246	1·53 (0·65-3·07)
Passau	247	0·76 (0·23-1·91)
Straubing	248	0·63 (0·17-1·63)
Deggendorf, Landkreis	249	3·76 (1·99-6·48)
Freyung-Grafenau, Landkreis	250	1·43 (0·58-2·95)
Kelheim, Landkreis	251	0·71 (0·31-1·4)
Landshut, Landkreis	252	1·98 (0·96-3·63)
Passau, Landkreis	253	5·17 (2·72-8·93)
Regen, Landkreis	254	4·53 (2·36-7·91)
Rottal-Inn, Landkreis	255	4·97 (2·66-8·49)
Straubing-Bogen, Landkreis	256	1·34 (0·62-2·55)
Dingolfing-Landau, Landkreis	257	2·61 (1·3-4·67)
Amberg	258	0·25 (0·03-0·89)
Regensburg	259	0·28 (0·11-0·62)
Weiden i.d.OPf.	260	0·13 (0·01-0·6)
Amberg-Sulzbach, Landkreis	261	0·37 (0·14-0·82)
Cham, Landkreis	262	1·29 (0·6-2·45)
Neumarkt i.d.OPf., Landkreis	263	0·51 (0·22-1·03)
Neustadt a.d.Waldnaab, Landkreis	264	0·29 (0·09-0·71)
Regensburg, Landkreis	265	0·43 (0·19-0·85)
Schwandorf, Landkreis	266	0·46 (0·18-0·95)
Tirschenreuth, Landkreis	267	0·35 (0·09-0·94)
Bamberg	268	1·59 (0·75-2·96)
Bayreuth	269	0·49 (0·16-1·14)
Coburg	270	15·43 (8·25-26·37)
Hof	271	0·33 (0·06-1·02)

Bamberg, Landkreis	272	1·6 (0·81-2·86)
Bayreuth, Landkreis	273	0·49 (0·21-0·98)
Coburg, Landkreis	274	11·23 (6·31-18·5)
Forchheim, Landkreis	275	0·95 (0·44-1·8)
Hof, Landkreis	276	0·35 (0·13-0·76)
Kronach, Landkreis	277	1·43 (0·67-2·69)
Kulmbach, Landkreis	278	0·76 (0·28-1·68)
Lichtenfels, Landkreis	279	2·09 (0·99-3·89)
Wunsiedel i.Fichtelgebirge, Landkreis	280	0·16 (0·03-0·47)
Ansbach	281	0·74 (0·22-1·85)
Erlangen	282	0·43 (0·18-0·89)
Fürth	283	1·66 (0·83-2·99)
Nürnberg	284	0·87 (0·45-1·52)
Schwabach	285	0·7 (0·24-1·63)
Ansbach, Landkreis	286	0·52 (0·23-1)
Erlangen-Höchstadt, Landkreis	287	0·57 (0·25-1·11)
Fürth, Landkreis	288	2·18 (1·09-3·89)
Nürnberger Land, Landkreis	289	0·71 (0·34-1·31)
Neustadt a.d.Aisch-Bad Windsheim, Landkreis	290	0·61 (0·24-1·29)
Roth, Landkreis	291	0·97 (0·45-1·85)
Weißenburg-Gunzenhausen, Landkreis	292	0·32 (0·1-0·77)
Aschaffenburg	293	0·54 (0·18-1·24)
Schweinfurt	294	0·47 (0·12-1·28)
Würzburg	295	0·97 (0·46-1·8)
Aschaffenburg, Landkreis	296	0·6 (0·28-1·11)
Bad Kissingen, Landkreis	297	1·29 (0·57-2·53)
Rhön-Grabfeld, Landkreis	298	0·99 (0·43-1·97)
Haßberge, Landkreis	299	1·63 (0·75-3·1)
Kitzingen, Landkreis	300	1·67 (0·81-3·06)
Miltenberg, Landkreis	301	1·85 (0·97-3·21)
Main-Spessart, Landkreis	302	1 (0·48-1·83)
Schweinfurt, Landkreis	303	1·11 (0·51-2·13)
Würzburg, Landkreis	304	2·13 (1·13-3·66)
Augsburg	305	1·91 (0·97-3·37)
Kaufbeuren	306	0·94 (0·31-2·22)
Kempten (Allgäu)	307	1 (0·36-2·21)
Memmingen	308	0·4 (0·12-0·99)
Aichach-Friedberg, Landkreis	309	3·7 (2·02-6·23)
Augsburg, Landkreis	310	1·37 (0·71-2·39)
Dillingen a.d.Donau, Landkreis	311	0·41 (0·15-0·92)
Günzburg, Landkreis	312	0·52 (0·21-1·05)
Neu-Ulm, Landkreis	313	0·49 (0·22-0·95)
Lindau (Bodensee), Landkreis	314	0·96 (0·41-1·95)
Ostallgäu, Landkreis	315	1·51 (0·73-2·79)
Unterallgäu, Landkreis	316	0·85 (0·4-1·58)

Donau-Ries, Landkreis	317	0·84 (0·4-1·56)
Oberallgäu, Landkreis	318	2·08 (1·03-3·74)
Saarbrücken, Regionalverband	319	0·94 (0·47-1·67)
Merzig-Wadern, Landkreis	320	0·77 (0·31-1·62)
Neunkirchen, Landkreis	321	0·3 (0·1-0·71)
Saarlouis, Landkreis	322	0·34 (0·14-0·7)
Saarpfalz-Kreis	323	0·53 (0·21-1·11)
St. Wendel, Landkreis	324	0·43 (0·15-1)
Berlin	325	5·02 (2·59-8·8)
Brandenburg an der Havel, Kreisfreie Stadt	326	0·8 (0·2-2·15)
Cottbus, Kreisfreie Stadt	327	0·55 (0·17-1·33)
Frankfurt (Oder), Kreisfreie Stadt	328	0·95 (0·27-2·4)
Potsdam, Kreisfreie Stadt	329	2·4 (1·07-4·65)
Barnim, Landkreis	330	2·55 (1·1-5·06)
Dahme-Spreewald, Landkreis	331	1·37 (0·57-2·79)
Elbe-Elster, Landkreis	332	0·4 (0·12-1·02)
Havelland, Landkreis	333	2·32 (1-4·62)
Märkisch-Oderland, Landkreis	334	1·17 (0·45-2·5)
Oberhavel, Landkreis	335	2·78 (1·28-5·3)
Oberspreewald-Lausitz, Landkreis	336	0·54 (0·17-1·3)
Oder-Spree, Landkreis	337	3·64 (1·7-6·87)
Ostprignitz-Ruppin, Landkreis	338	1·97 (0·82-4·02)
Potsdam-Mittelmark, Landkreis	339	4·32 (2·03-8·09)
Prignitz, Landkreis	340	0·7 (0·19-1·82)
Spree-Neiße, Landkreis	341	0·8 (0·27-1·84)
Teltow-Fläming, Landkreis	342	1·53 (0·62-3·19)
Uckermark, Landkreis	343	0·74 (0·26-1·7)
Kreisfreie Stadt Rostock, Hansestadt	344	0·23 (0·06-0·62)
Kreisfreie Stadt Schwerin, Landeshauptstadt	345	0·29 (0·07-0·83)
Landkreis Mecklenburgische Seenplatte	346	0·54 (0·23-1·08)
Landkreis Rostock	347	0·21 (0·06-0·52)
Landkreis Vorpommern-Rügen	348	0·3 (0·08-0·77)
Landkreis Nordwestmecklenburg	349	0·45 (0·15-1·06)
Landkreis Vorpommern-Greifswald	350	0·56 (0·22-1·2)
Landkreis Ludwigslust-Parchim	351	0·64 (0·28-1·28)
Chemnitz, Stadt	352	1·97 (0·89-3·78)
Erzgebirgskreis	353	0·31 (0·1-0·73)
Mittelsachsen, Landkreis	354	1·43 (0·66-2·72)
Vogtlandkreis	355	0·72 (0·3-1·47)
Zwickau, Landkreis	356	1·21 (0·52-2·4)
Dresden, Stadt	357	2·2 (1·06-4·06)
Bautzen, Landkreis	358	0·78 (0·33-1·56)
Görlitz, Landkreis	359	0·27 (0·07-0·71)
Meißen, Landkreis	360	0·6 (0·24-1·26)
Sächsische Schweiz-Osterzgebirge, Landkreis	361	0·78 (0·3-1·65)

Leipzig, Stadt	362	2·41 (1·16-4·44)
Leipzig, Landkreis	363	0·57 (0·21-1·22)
Nordsachsen, Landkreis	364	0·36 (0·13-0·82)
Dessau-Roßlau, Kreisfreie Stadt	365	0·37 (0·07-1·14)
Halle (Saale), Kreisfreie Stadt	366	3·34 (1·56-6·29)
Magdeburg, Kreisfreie Stadt	367	0·47 (0·16-1·07)
Altmarkkreis Salzwedel	368	3·39 (1·55-6·48)
Anhalt-Bitterfeld, Landkreis	369	0·42 (0·14-0·98)
Börde, Landkreis	370	0·46 (0·17-1·03)
Burgenlandkreis	371	1·37 (0·59-2·73)
Harz, Landkreis	372	0·44 (0·16-0·97)
Jerichower Land, Landkreis	373	1·27 (0·46-2·83)
Mansfeld-Südharz, Landkreis	374	0·37 (0·1-0·95)
Saalekreis	375	1·1 (0·46-2·25)
Salzlandkreis	376	0·37 (0·13-0·84)
Stendal, Landkreis	377	0·62 (0·21-1·4)
Wittenberg, Landkreis	378	1·3 (0·51-2·74)
Erfurt, krsfr. Stadt	379	3·7 (1·77-6·83)
Gera, krsfr. Stadt	380	1·26 (0·41-2·96)
Jena, krsfr. Stadt	381	1·25 (0·51-2·58)
Suhl, krsfr. Stadt	382	3·54 (1·4-7·46)
Weimar, krsfr. Stadt	383	5·92 (2·65-11·48)
Eisenach, krsfr. Stadt	384	0·6 (0·14-1·7)
Eichsfeld, Kreis	385	6·47 (2·95-12·37)
Nordhausen, Kreis	386	0·48 (0·15-1·18)
Wartburgkreis	387	0·48 (0·17-1·07)
Unstrut-Hainich-Kreis	388	1·04 (0·38-2·3)
Kyffhäuserkreis	389	1·07 (0·37-2·43)
Schmalkalden-Meiningen, Kreis	390	0·76 (0·29-1·66)
Gotha, Kreis	391	0·9 (0·35-1·91)
Sömmerda, Kreis	392	1·45 (0·51-3·26)
Hildburghausen, Kreis	393	2·05 (0·9-4·03)
Ilm-Kreis	394	0·78 (0·29-1·7)
Weimarer Land, Kreis	395	2·21 (0·92-4·48)
Sonneberg, Kreis	396	1·75 (0·65-3·81)
Saalfeld-Rudolstadt, Kreis	397	0·7 (0·25-1·55)
Saale-Holzland-Kreis	398	1·05 (0·38-2·34)
Saale-Orla-Kreis	399	0·72 (0·26-1·63)
Greiz, Kreis	400	0·99 (0·36-2·19)
Altenburger Land, Kreis	401	0·49 (0·13-1·28)

Table S7: ARR exceedance > 2 with posterior probability greater than or equal to 80 %

District (n = 54)	Area ID	ARR (95%-CrI)	Posterior probability of ARR > 2
Neumünster, Kreisfreie Stadt	4	3·73 (1·63-7·36)	0.9218
Segeberg, Landkreis	13	2·87 (1·37-5·34)	0.8059
Lüchow-Dannenberg, Landkreis	37	4·78 (2·33-8·74)	0.9917
Verden, Landkreis	44	6·05 (3·02-10·86)	0.9993
Emden, Kreisfreie Stadt	46	8·4 (4·09-15·37)	1
Leer, Landkreis	56	6·76 (3·44-11·98)	0.9999
Düsseldorf, Kreisfreie Stadt	64	2·65 (1·47-4·42)	0.803
Duisburg, Kreisfreie Stadt	65	9·9 (5·77-15·86)	1
Essen, Kreisfreie Stadt	66	3·09 (1·77-5)	0.9331
Krefeld, Kreisfreie Stadt	67	2·94 (1·57-5·03)	0.8713
Mönchengladbach, Kreisfreie Stadt	68	3·86 (2·14-6·43)	0.9856
Mülheim an der Ruhr, Kreisfreie Stadt	69	2·98 (1·58-5·12)	0.8786
Oberhausen, Kreisfreie Stadt	70	3·11 (1·63-5·41)	0.8997
Mettmann, Kreis	75	2·62 (1·49-4·29)	0.8067
Wesel, Kreis	78	4·14 (2·37-6·71)	0.9952
Leverkusen, Kreisfreie Stadt	81	2·78 (1·44-4·84)	0.8157
Euskirchen, Kreis	85	4·11 (2·32-6·75)	0.9938
Oberbergischer Kreis	87	3·42 (1·94-5·58)	0.9677
Rheinisch-Bergischer Kreis	88	3·18 (1·77-5·26)	0.9362
Offenbach am Main, Kreisfreie Stadt	119	7·23 (3·85-12·41)	1
Wetteraukreis	130	3·68 (2·06-6·08)	0.9802
Gießen, Landkreis	131	2·81 (1·48-4·84)	0.8329
Lahn-Dill-Kreis	132	2·95 (1·54-5·14)	0.8653
Altenkirchen (Westerwald), Landkreis	145	2·88 (1·5-5·02)	0.8448
Cochem-Zell, Landkreis	148	3·72 (1·79-6·88)	0.9481
Vulkaneifel, Landkreis	157	5·52 (2·95-9·43)	0.9994
Ortenaukreis, Landkreis	207	3·2 (1·77-5·34)	0.9354
München, Landeshauptstadt	224	2·73 (1·5-4·56)	0.8286
Berchtesgadener Land, Landkreis	227	3·87 (1·99-6·82)	0.9732
Bad Tölz-Wolfratshausen, Landkreis	228	3·41 (1·79-5·91)	0.9434
Dachau, Landkreis	229	2·8 (1·47-4·85)	0.8271
Fürstenfeldbruck, Landkreis	234	3·27 (1·73-5·64)	0.9294
Garmisch-Partenkirchen, Landkreis	235	5·15 (2·71-8·91)	0.9984
Landsberg am Lech, Landkreis	236	4·37 (2·31-7·53)	0.9925
Mühldorf a.Inn, Landkreis	238	4·3 (2·3-7·35)	0.9921
Rosenheim, Landkreis	242	3·05 (1·65-5·16)	0.9021
Starnberg, Landkreis	243	3·33 (1·74-5·8)	0.9324
Weilheim-Schongau, Landkreis	245	4·55 (2·41-7·82)	0.995
Deggendorf, Landkreis	249	3·76 (1·99-6·48)	0.9734
Passau, Landkreis	253	5·17 (2·72-8·93)	0.9985
Regen, Landkreis	254	4·53 (2·36-7·91)	0.9934

Rottal-Inn, Landkreis	255	4·97 (2·66-8·49)	0.9982
Coburg	270	15·43 (8·25-26·37)	1
Coburg, Landkreis	274	11·23 (6·31-18·5)	1
Aichach-Friedberg, Landkreis	309	3·7 (2·02-6·23)	0.9762
Berlin	325	5·02 (2·59-8·8)	0.9972
Oder-Spree, Landkreis	337	3·64 (1·7-6·87)	0.9324
Potsdam-Mittelmark, Landkreis	339	4·32 (2·03-8·09)	0.9768
Halle (Saale), Kreisfreie Stadt	366	3·34 (1·56-6·29)	0.8957
Altmarkkreis Salzwedel	368	3·39 (1·55-6·48)	0.8965
Erfurt, krsfr. Stadt	379	3·7 (1·77-6·83)	0.9454
Suhl, krsfr. Stadt	382	3·54 (1·4-7·46)	0.8681
Weimar, krsfr. Stadt	383	5·92 (2·65-11·48)	0.9965
Eichsfeld, Kreis	385	6·47 (2·95-12·37)	0.9987

Table S8: ARR exceedance > 3 with posterior probability greater than or equal to 80 %

District (n = 22)	Area ID	ARR (95%-CrI)	Posterior probability of ARR > 2
Lüchow-Dannenberg, Landkreis	37	4·78 (2·33-8·74)	0.8857
Verden, Landkreis	44	6·05 (3·02-10·86)	0.9758
Emden, Kreisfreie Stadt	46	8·4 (4·09-15·37)	0.9979
Leer, Landkreis	56	6·76 (3·44-11·98)	0.9914
Duisburg, Kreisfreie Stadt	65	9·9 (5·77-15·86)	1
Wesel, Kreis	78	4·14 (2·37-6·71)	0.8577
Euskirchen, Kreis	85	4·11 (2·32-6·75)	0.8454
Offenbach am Main, Kreisfreie Stadt	119	7·23 (3·85-12·41)	0.9973
Vulkaneifel, Landkreis	157	5·52 (2·95-9·43)	0.9711
Garmisch-Partenkirchen, Landkreis	235	5·15 (2·71-8·91)	0.947
Landsberg am Lech, Landkreis	236	4·37 (2·31-7·53)	0.8628
Mühldorf a.Inn, Landkreis	238	4·3 (2·3-7·35)	0.8543
Weilheim-Schongau, Landkreis	245	4·55 (2·41-7·82)	0.8906
Passau, Landkreis	253	5·17 (2·72-8·93)	0.9489
Regen, Landkreis	254	4·53 (2·36-7·91)	0.8794
Rottal-Inn, Landkreis	255	4·97 (2·66-8·49)	0.9393
Coburg	270	15·43 (8·25-26·37)	1
Coburg, Landkreis	274	11·23 (6·31-18·5)	1
Berlin	325	5·02 (2·59-8·8)	0.931
Potsdam-Mittelmark, Landkreis	339	4·32 (2·03-8·09)	0.8015
Weimar, krsfr. Stadt	383	5·92 (2·65-11·48)	0.9478
Eichsfeld, Kreis	385	6·47 (2·95-12·37)	0.9717
