# Supplementary material for: Socioeconomic deprivation is inversely associated with measles incidence: a longitudinal small-area analysis, Germany, 2001 to 2017
Source: Euro Surveill. 2021 Apr 29;26(17):1900755. doi: 10.2807/1560-7917.ES.2021.26.17.1900755 (PMC8086244; doi:10.2807/1560-7917.ES.2021.26.17.1900755)
Supplement: Supplement S1 [file 1900755_Interactive_Map.zip › Eurosurveillance_Disclaimer.pdf]

This supplementary material is hosted by *Eurosurveillance* as supporting information alongside the article “Socioeconomic deprivation is inversely associated with measles incidence: a longitudinal small-area analysis, Germany, 2001 to 2017”, on behalf of the authors, who remain responsible for the accuracy and appropriateness of the content. The same standards for ethics, copyright, attributions and permissions as for the article apply. Supplements are not edited by *Eurosurveillance* and the journal is not responsible for the maintenance of any links or email addresses provided therein."
