# Supplementary material for: Socioeconomic deprivation is inversely associated with measles incidence: a longitudinal small-area analysis, Germany, 2001 to 2017
Source: Euro Surveill. 2021 Apr 29;26(17):1900755. doi: 10.2807/1560-7917.ES.2021.26.17.1900755 (PMC8086244; doi:10.2807/1560-7917.ES.2021.26.17.1900755)
Supplement: Supplement S1 [file 1900755_Interactive_Map.zip › MAP.zip!/MAP/Measles_Map_int.html]

leaflet
